# Telemedicine-Based Management of Oral Anticoagulation Therapy: Systematic Review and Meta-analysis

**DOI:** 10.2196/45922

**Published:** 2023-07-10

**Authors:** Letícia Braga Ferreira, Rodrigo Lanna de Almeida, Alair Arantes, Hebatullah Abdulazeem, Ishanka Weerasekara, Leticia Santos Dias Norberto Ferreira, Luana Fonseca de Almeida Messias, Luciana Siuves Ferreira Couto, Maria Auxiliadora Parreiras Martins, Núbia Suelen Antunes, Raissa Carolina Fonseca Cândido, Samuel Rosa Ferreira, Tati Guerra Pezzini Assis, Thais Marques Pedroso, Eric Boersma, Antonio Ribeiro, Milena Soriano Marcolino

**Affiliations:** 1 University Hospital Universidade Federal de Minas Gerais Belo Horizonte Brazil; 2 Rede Mater Dei de Saúde Belo Horizonte Brazil; 3 Laboratory of Respiratory Physiology University of Brasilia Brasilia Brazil; 4 Department of Sport and Health Sciences Technical University of Munich Munich Germany; 5 Faculty of Health and Social Sciences Western Norway University of Applied Sciences Bergen Norway; 6 School of Health Sciences College of Health, Medicine and Wellbeing University of Newcastle Callaghan Australia; 7 Medical School Universidade Federal de Ouro Preto Ouro Preto Brazil; 8 Faculty of Pharmacy Universidade Federal de Minas Gerais Belo Horizonte Brazil; 9 Emergency Department Fundação Hospitalar do Estado de Minas Gerais Belo Horizonte Brazil; 10 Santa Casa de Belo Horizonte Belo Horizonte Brazil; 11 Thoraxcenter Erasmus Medical Center Rotterdam Netherlands; 12 Institute for Health Technology Assessment Porto Alegre Brazil

**Keywords:** anticoagulation, telemedicine, eHealth, warfarin, DOACs, atrial fibrillation

## Abstract

**Background:**

Oral anticoagulation is the cornerstone treatment of several diseases. Its management is often challenging, and different telemedicine strategies have been implemented to support it.

**Objective:**

The aim of the study is to systematically review the evidence on the impact of telemedicine-based oral anticoagulation management compared to usual care on thromboembolic and bleeding events.

**Methods:**

Randomized controlled trials were searched in 5 databases from inception to September 2021. Two independent reviewers performed study selection and data extraction. Total thromboembolic events, major bleeding, mortality, and time in therapeutic range were assessed. Results were pooled using random effect models.

**Results:**

In total, 25 randomized controlled trials were included (n=25,746 patients) and classified as moderate to high risk of bias by the Cochrane tool. Telemedicine resulted in lower rates of thromboembolic events, though not statistically significant (n=13 studies, relative risk [RR] 0.75, 95% CI 0.53-1.07; *I*^2^=42%), comparable rates of major bleeding (n=11 studies, RR 0.94, 95% CI 0.82-1.07; *I*^2^=0%) and mortality (n=12 studies, RR 0.96, 95% CI 0.78-1.20; *I*^2^=11%), and an improved time in therapeutic range (n=16 studies, mean difference 3.38, 95% CI 1.12-5.65; *I*^2^=90%). In the subgroup of the multitasking intervention, telemedicine resulted in an important reduction of thromboembolic events (RR 0.20, 95% CI 0.08-0.48).

**Conclusions:**

Telemedicine-based oral anticoagulation management resulted in similar rates of major bleeding and mortality, a trend for fewer thromboembolic events, and better anticoagulation quality compared to standard care. Given the potential benefits of telemedicine-based care, such as greater access to remote populations or people with ambulatory restrictions, these findings may encourage further implementation of eHealth strategies for anticoagulation management, particularly as part of multifaceted interventions for integrated care of chronic diseases. Meanwhile, researchers should develop higher-quality evidence focusing on hard clinical outcomes, cost-effectiveness, and quality of life.

**Trial Registration:**

PROSPERO International Prospective Register of Systematic Reviews CRD42020159208; https://www.crd.york.ac.uk/prospero/display_record.php?RecordID=159208

## Introduction

Oral anticoagulation is the cornerstone treatment of several diseases and has been prescribed to millions worldwide. Atrial fibrillation (AF) and venous thromboembolism (VTE) are the most common indications, with AF prevalence estimated at 46.3 million people worldwide [1] and VTE incidence that varies from 115 to 269 per 100,000 population depending on the country [2].

Direct oral anticoagulants (DOACs) have progressively replaced vitamin K antagonists (VKAs) [3]. However, in certain conditions, especially antiphospholipid syndrome, mechanical heart valves, and rheumatic mitral stenosis, VKAs remain the only drugs with established safety and efficacy [4,5]. Additionally, in low-income contexts, they are frequently the preferred option due to the high costs of DOACs. Management of VKA therapy involves serial testing for the international normalized ratio (INR) value to guide dose adjustment. The quality of oral anticoagulation therapy (OAT), often expressed as time in therapeutic range (TTR), strongly correlates with the incidence of bleeding and thromboembolic events [6].

Different eHealth strategies have been implemented to support OAT management. Studies have usually focused on the impact of telemedicine on anticoagulation quality. Data on clinical outcomes are scarce due to the small number of patients enrolled or the short length of follow-up, both of which result in low event rates, often rendering the studies inconclusive [7-9]. Therefore, summarizing the best available evidence on the topic is necessary, especially in light of the substantial rise in telehealth use observed during the COVID-19 pandemic [10]. This study aimed to systematically review the evidence that assesses the impact of telemedicine-based OAT management compared to usual care on relevant outcomes.

## Methods

This systematic review was conducted in accordance with the *Cochrane Handbook for Systematic Reviews of Interventions, version 6.2* [11] and reported according to the PRISMA (Preferred Reporting Items for Systematic Reviews and Meta-Analyses) statement [12]. The protocol was registered on PROSPERO (CRD42020159208).

### Search Strategy

MEDLINE, Embase, Cochrane Library, and LILACS were searched for relevant studies in September 2021 with no time or language restrictions. Google Scholar was used as a gray literature source, and reference lists of the included studies were hand-searched for additional studies of interest. The complete search strategy is provided in Multimedia Appendix 1.

### Outcomes

Primary outcomes included the incidence of total thromboembolic events (TTEs; efficacy outcome) and major hemorrhagic events (safety outcome), as defined by each study, measured at any time point. Secondary outcomes were all-cause mortality and quality of anticoagulation (for VKA studies) measured by the TTR.

### Studies Selection

Two investigators independently screened the studies to include individual or cluster randomized controlled trials that bore comparisons between any telemedicine intervention and control groups of usual care for the management of adult outpatients on OAT for any condition.

Exclusion criteria were as follows: (1) trials that used any kind of telemedicine strategy in the control group; (2) studies not reported in full text; (3) in-hospital telemedicine intervention; (4) duplicate publications or substudies of included studies. In the latter case, we selected the publication with the largest sample and longest follow-up.

Disagreements were resolved by discussion with a third reviewer. Whenever necessary, we contacted corresponding authors to obtain data not included in the publication using email and Research Gate. Table 1 details the various types of telemedicine interventions included.

**Table 1:** Telemedicine categories.

Category of telemedicine intervention	Description
Computer-assisted dosing	Use of computerized algorithms for VKA^a^ dose adjustment
Laboratory testing with remote adjustment	Conventional laboratory testing for INR^b^ values and dose adjustment made by remote assistance (either by phone, fax, mobile app, or internet-based system)
Self-testing	Self-testing for INR values using point-of-care devices and dose adjustment either by remote assistance or self-management (with remote professional support)
Multitasking application	Mobile app or internet-based CDSS^c^ for atrial fibrillation care, including anticoagulation indication and management, rhythm or rate control, symptom control, and cardiovascular risk factors management

^a^VKA: vitamin K antagonist.

^b^INR: international normalized ratio.

^c^CDSS: clinical decision support system.

### Data Extraction and Quality Assessment

Data extraction and risk of bias analysis were independently performed by 2 investigators using the Cochrane risk of bias tool for randomized trials [13] and the Cochrane risk of bias for cluster-randomized trials [14]. The body of evidence’s overall quality was rated using the Grading of Recommendations Assessment, Development, and Evaluation approach [15].

### Data Synthesis and Analyses

Statistical analyses were performed with ReviewManager Software (RevMan, version 5.4.1; Cochrane) using random effect models. Mean differences (MDs) were calculated for continuous outcomes, and pooled relative risks (RRs) for binary outcomes with respective 95% CIs.

Data from cluster trials were pooled after adjusting for the intracluster effect. When adjusted data were not provided in the original publication or after contact with the study authors, we adjusted it using intracluster correlation coefficient values obtained from external studies with similar populations.

Statistical heterogeneity of the treatment effect among studies was investigated using the *I*^2^ statistic. The funnel plot, Egger’s test, and the Trim and Fill method were used to investigate publication bias and were calculated using the Meta-essentials worksheet [16].

Sensitivity analyses were conducted by excluding each individual study at a time, excluding studies with a high risk of bias, and adjusting cluster trial data using different intracluster correlation coefficient values. Subgroup analyses were carried out for different modalities of telemedicine intervention.

## Results

### Search Results and Study Selection

**Figure 1:**
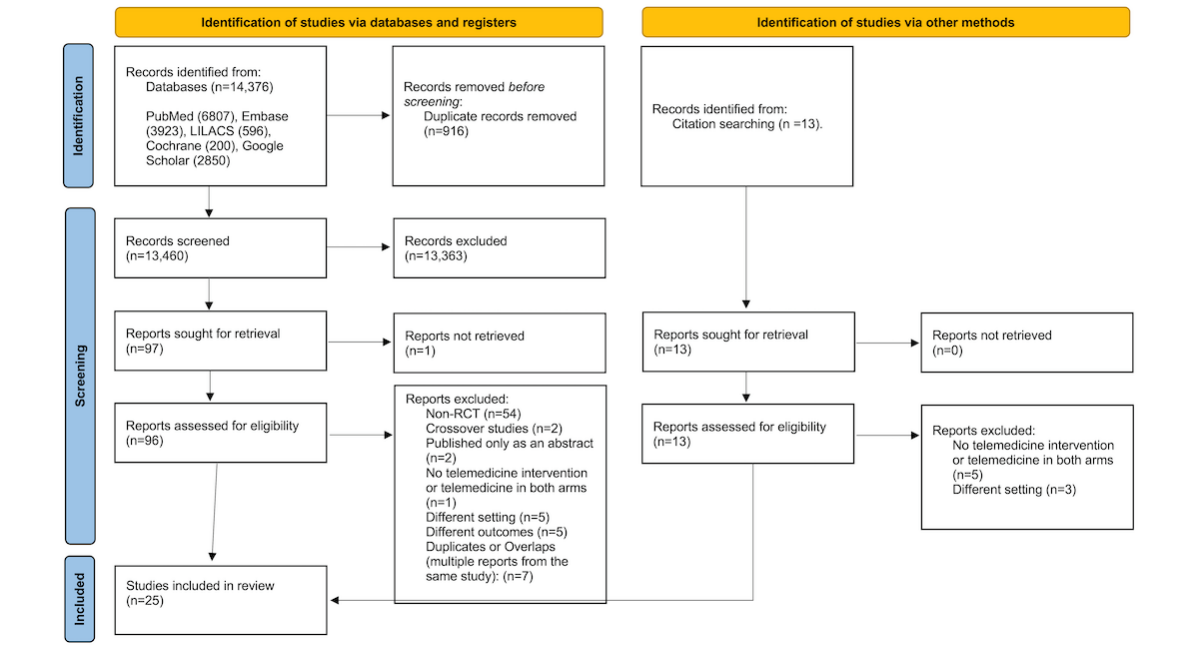
PRISMA (Preferred Reporting Items for Systematic Reviews and Meta-Analyses) flow diagram. RCT: randomized controlled trial.

The electronic search identified 14,376 records. We removed 916 duplicates and screened 13,460 titles. Another 13 records were identified by a manual search of the reference lists. After title and abstract screening, 109 full texts were retrieved. Of these, 84 did not meet the inclusion criteria and were excluded; thus, 25 papers were included (Figure 1).

### Studies and Patients’ Characteristics

The 25 studies included 3 cluster randomized controlled trials [17-19] and 22 individually randomized parallel-group trials [7-9,20-38], totaling 25,746 patients. One study held 2 independent comparisons based on different INR target ranges, so these distinct pairs of comparisons are represented as “Vadher a” and “Vadher b” in some analyses [22]. Table 2 describes the main characteristics of the included studies.

AF was the most prevalent indication for anticoagulation (n=12,448, 55.3% of patients with known indication) followed by VTE (n=3842, 16.0%) and valvular heart disease (n=3701, 15.7%). Most studies enrolled patients using VKAs. Patients receiving DOAC were included in 2 studies: they made up 29% (n=329) of patients in the Cox trial and 60% (n=1484) of patients in the Guo trial. Only 4 studies had a mean follow-up period of more than a year.

Different types of telemedicine interventions were tested across the included studies. In 11 studies [17,20-22,25-29,31,35], the telemedicine intervention was mainly based on the use of clinical decision support system for VKA dose adjustment or scheduling of the next visit. Overall, 12 studies [7-9,23,24,30,32-34,36-38] involved some kind of remote support (either by telephone, mobile app, or internet-based systems) for VKA dose adjustment—8 used self-testing with point-of-care devices for INR measurement, and 4 used conventional laboratory testing. Two studies [18,19] assessed the impact of a multitasking intervention (via a mobile app or a web-based clinical decision support system) for the management of AF in primary care, which included anticoagulation therapy indication and management, along with rate or rhythm control, symptom monitoring, and other cardiovascular risk factors management.

Most studies used the Rosendaal method to calculate TTR [39]. Four studies made cost analyses [9,17,24,27], which are qualitatively described in Multimedia Appendix 1.

**Table 2:** Characteristics of included studies.

Authors, publication year	Registration number	Value, n	Age (years), mean	% male sex	Indications for anticoagulation	Description of the intervention	Drug	Follow-up (months), mean	Primary outcome	Thromboembolic events: intervention (%)/ control (%)	Major bleeding: intervention (%)/ control (%)	Mortality: intervention (%)/ control (%)	TTR^a^: intervention (%)/ control (%)
Ageno et al (1998) [32]	N/A^b^	101	52	N/A	Valvular heart disease: 100%	Computer-assisted algorithm for anticoagulant dose adjustment made by phone or fax	Warfarin	10	Average number of INR^c^ tests	N/A	N/A	N/A	55.2/ 55.3
Ayutthaya et al (2018) [7]	TCTR20180614006	50	57.6	40	AF^d^: 62% DVT^e^/PE^f^: 30% Valvular heart disease: 34%	Telephone follow-up by pharmacists	Warfarin	3	TTR (Rosendaal method), number of patients with out-of-range INR	12/ 24	8/4	0/4	49.8/ 28
Borgman et al (2012) [28]	NCT00993200	26	53	54	AF: 34.5% DVT: 46% Cerebrovascular disease: 7%	Computer-assisted algorithm for anticoagulant dose adjustment (genotype information added)	Warfarin	3	Time to first stable therapeutic INR	N/A	N/A	N/A	77.7/ 70.3
Christensen et al (2011) [30]	N/A	123	N/A	74.8	AF: 53.4% Cerebrovascular disease: 9.7% DVT/PE: 16.3% Valvular heart disease: 12.5% Others: 13.2%	Self-testing and dose adjustments made through a web-based system	Warfarin	11	TTR (Rosendaal method)	N/A	N/A	0/0	79.9/ 72.7
Cox et al (2020) [19]	NCT01927367	1133	72.3	61.8	AF: 100%	Web-based, point-of-care CDSS^g^ designed to enable rapid, evidence-based treatment of AF	Warfarin and DOAC^h^ (29%)	12	AF-related emergency department visit or unplanned cardiovascular hospitalization, and major bleeding	N/A	1.5/ 1.2	4.7/ 3.8	N/A
Fihn et al (1994) [20]	N/A	620	61	71	AF: 17% Cerebrovascular disease: 10% Systemic embolism: 6% DVT/PE: 26% Others: 42%	Computer-generated recommendation for scheduling the next visit	Warfarin	8	Follow-up interval scheduled and the quality of anticoagulation control	1.9/ 0.9	4.3/ 4.7	0/0	N/A
Fitzmaurice et al (1996) [27]	N/A	23	N/A	N/A	AF: 13% Systemic embolism: 4.3% DVT/PE: 43.4% Valvular heart disease: 30.4% Others: 8.6%	Computer-assisted algorithm for anticoagulant dose adjustment	Warfarin	12	Percentage of INR results in therapeutic range	7.1/0	0/0	7.1/ 14.3	N/A
Fitzmaurice et al (2000) [17]	N/A	367	N/A	55	AF: 48% Cerebrovascular disease: 4% Systemic embolism: 2% DVT/PE: 20% Valvular heart disease: 16%	Near-patient testing and computer-assisted algorithm for anticoagulant dose adjustment	Warfarin	12	INR control (point prevalence of patients achieving individual therapeutic INR targets) and TTR	1.6/4	0/0	2.4/ 2.5	69/ 62
Fitzmaurice et al (2002) [9]	N/A	49	65	75.5	AF: 55.1% Others: 44.9%	Self-testing and self-management of anticoagulation. Remote support available through pager and telephone	Warfarin	6	TTR (method not mentioned)	N/A	0/3.8	0/3.8	74/ 77
Gadisseur et al (2003) [23]	N/A	320	58.5	71.3	AF: 21.3% Cerebrovascular disease: 1.3% Systemic embolism: 2.2% DVT/PE: 20.3% Valvular heart disease: 19.1% Others: 35.6%	Self-testing with or without self-management of OAT^i^ by patients (phone support by the anticoagulation clinic)	Phenprocoumon and acenocoumarol	6	Quality of anticoagulation therapy; thromboembolic and hemorrhagic events	0/0	2/1.4	N/A	67.7/ 64.7
Guo et al (2020) [40]	ChiCTR-OOC-17014138	2473	68.9	62	AF: 100%	Use of mobile app for integrated management of AF, including anticoagulation management	VKAs^j^ and DOACs (60%)	20	Composite of stroke, thromboembolism, all-cause death, and rehospitalization	0.8/5	0/0.4	0.9/ 2.6	N/A
Khan et al (2004) [37]	N/A	79	73	60	AF: 100%	Self-testing and dose adjustment made by physician by telephone	Warfarin	6	TTR (Rosendaal method)	N/A	N/A	N/A	71.1/ 70.4
Manotti et al (2001) [35]	N/A	1251	67.1	54.8	Systemic embolism: 26% DVT/PE: 21.2% Valvular heart disease: 14.6% Others: 38.1%	Computer-assisted algorithm for anticoagulant dose adjustment	Warfarin and acenocoumarol	8.1	Percentage of patients reaching a stable INR and TTR (Rosendaal method)	N/A	N/A	N/A	71.2/ 68.2
Matchar et al (2010) [24]	NCT00032591	2922	67	98	AF: 76.5% Valvular heart disease: 23.4%	Self-testing and dose adjustments made by phone, after communication of results using interactive voice-response system	Warfarin	36	Time to first major event (stroke, major bleeding episode, or death)	4.8/ 5.6	12.2/ 13.6	10.3/ 10.7	66.2/ 62.4
Nieuwlaat et al (2012) [25]	NCT01024452	1298	68.6	62.3	AF: 47.7% Cerebrovascular disease: 3.6% Valvular heart disease: 25.1% DVT/PE: 15.8% Others: 7.9%	Computer-assisted algorithm for anticoagulant dose adjustment and scheduling the next visit	Warfarin	5.3	TTR (Rosendaal method)	N/A	N/A	N/A	71/ 71.9
Poller et al (1993) [31]	N/A	186	64.5	57.5	AF: 12.4% Cerebrovascular disease: 5.4% Systemic embolism: 30.1% Valvular heart disease: 8.1% DVT/PE: 39.8% Others: 4.3%	Computer-assisted algorithm for anticoagulant dose adjustment	Warfarin	6	Binary indicator for out-of-range INR	N/A	0/0	0.8/0	N/A
Poller et al (1998) [26]	N/A	285	N/A	N/A	N/A	Computer-assisted algorithm for anticoagulant dose adjustment	Warfarin and acenocoumarol	N/A	TTR (Rosendaal method)	N/A	N/A	N/A	63.3/ 53.2
Poller et al (2008) [21]	N/A	13,052	66.9	53.5	AF: 45.6% DVT/PE: 24.5% Valvular heart disease: 13% Others: 16.8%	Computer-assisted algorithm for anticoagulant dose adjustment	Warfarin, acenocoumarol, and phenprocoumon	N/A	Incidence of clinical events (bleeding or thrombotic)	1.4/ 1.6	1.4/ 1.5	1.1/ 0.9	65.9/ 64.7
Rasmussen et al (2012) [29]	N/A	54	70	57	N/A	Computer-assisted algorithm for anticoagulant dose adjustment	Warfarin	7	TTR (Rosendaal method)	N/A	N/A	N/A	53.1/ 55
Sidhu and O’Kane (2001) [8]	N/A	100	60.9	46	Valvular heart disease: 100%	Self-testing and self-management of anticoagulation. Physician available remotely for doubts	Warfarin	24	Number of tests in therapeutic range and TTR (Rosendaal method)	21.9/ 22.9	2.4/0	0/8.3	76.6/ 63.8
Staresinic et al (2006) [33]	N/A	192	69.3	97.4	AF: 41.1% Stroke: 9.9% DVT/PE: 12% Valvular heart disease: 18.8% Others: 18.2%	Laboratory testing of INR. dose adjustment and counseling made by phone contact by the clinic staff	Warfarin	36	TTR (Rosendaal method)	4/9.5	49/ 45.7	13.2/ 9.5	57.8/ 55.1
Vadher et al (1997) [22]	N/A	177	62.9	56.5	N/A	Computer-assisted algorithm for anticoagulant dose adjustment	Warfarin	N/A	TTR (Rosendaal method)	5.4/ 2.2	N/A	N/A	60.7/ 51.6
Verret et al (2012) [38]	NCT01033279	114	57.7	68	AF: 50.8% Valvular heart disease: 42.1% Others: 7%	Self-testing and self-management of anticoagulation. Pharmacist supervision through voicemail messages and telephone contact	Warfarin	4	Anticoagulation-related quality of life	0/0	3.4/ 1.7	0/0	N/A
Vogeler et al (2021) [34]	N/A	30	61	93.5	LVAD^k^: 100%	Self-testing, results transmitted via telemedicine device, and remote dose adjustment by clinic staff	Warfarin and phenprocoumon	12	TTR (Rosendaal method)	26/ 6.6	N/A	N/A	58/ 78
Zhu et al (2021) [36]	ChiCTR1800016204	721	50.1	61	Valvular heart disease: 100%	Internet-based anticoagulation management via a mobile user interface medical platform	Warfarin	12	TTR (Rosendaal method)	0.2/ 0.5	0.5/ 1.1	0/0.5	53/ 46

^a^TTR: time in therapeutic range.

^b^N/A: not available.

^c^INR: international normalized ratio.

^d^AF: atrial fibrillation.

^e^DVT: deep venous thrombosis.

^f^PE: pulmonary embolism.

^g^CDSS: clinical decision support system.

^h^DOAC: direct oral anticoagulant.

^i^OAT: oral anticoagulation therapy.

^j^VKA: vitamin K antagonist.

^k^LVAD: left ventricular assist device.

### Risk of Bias

**Figure 2:**
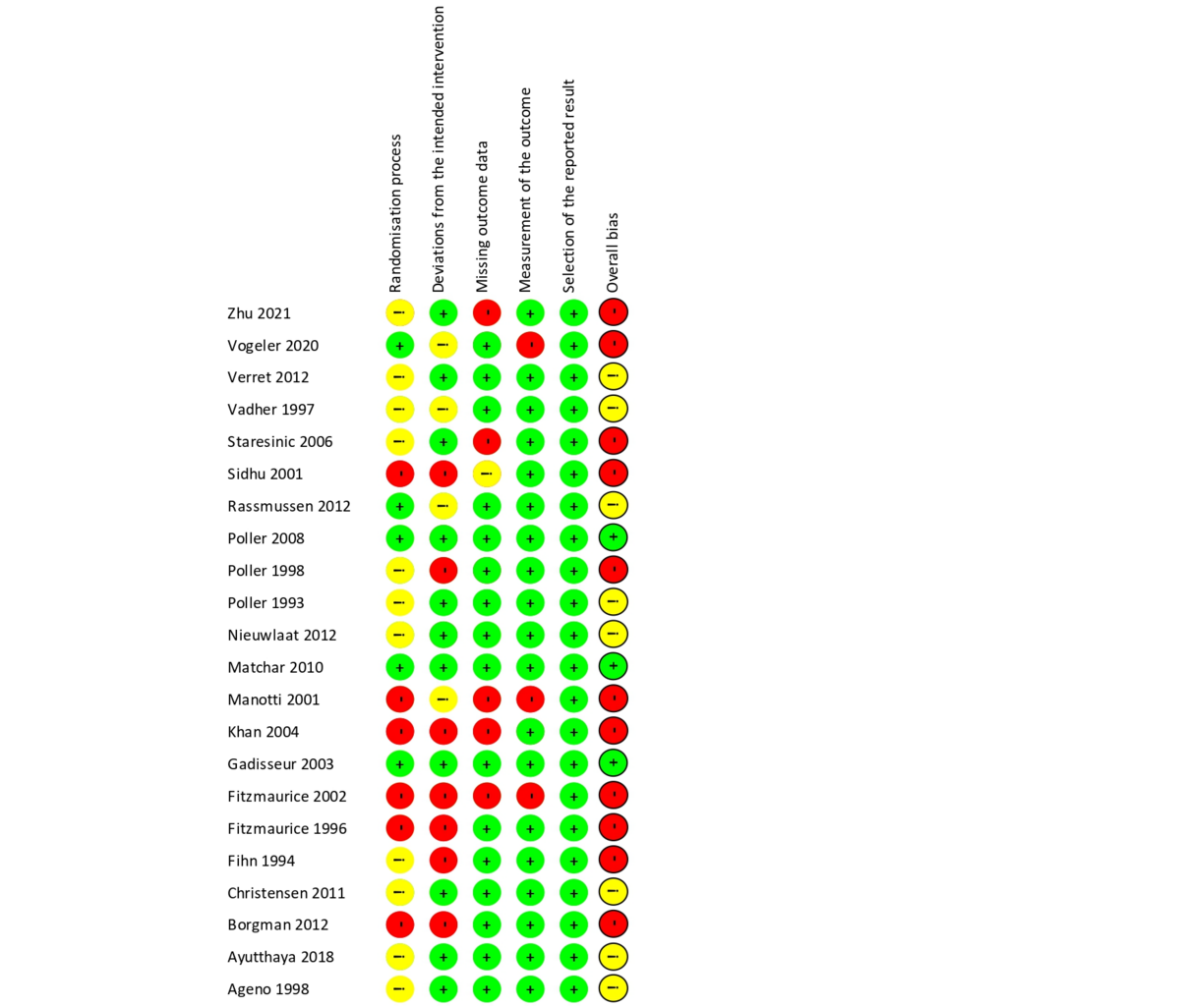
Risk of bias in individual randomized studies [7-9,20-38]. Green: low risk of bias; Red: high risk of bias; Yellow: unclear risk of bias.

**Figure 3:**
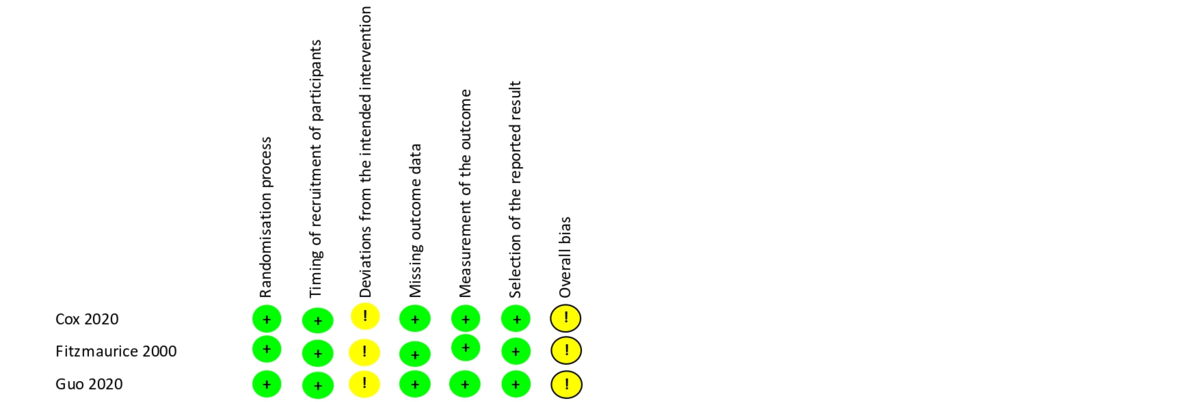
Risk of bias in cluster randomized studies [17,19,41]. Green: low risk of bias; Yellow: unclear risk of bias.

Risk of bias in included studies is shown in Figures 2 and 3. Only 3 studies were considered to have a low risk of bias. No study was double-blinded, which could have caused deviations from the intended interventions in 7 trials. The randomization process was poorly described in 17 studies, and missing relevant outcome data were detected in 5 studies.

### Primary and Secondary Outcomes

**Figure 4:**
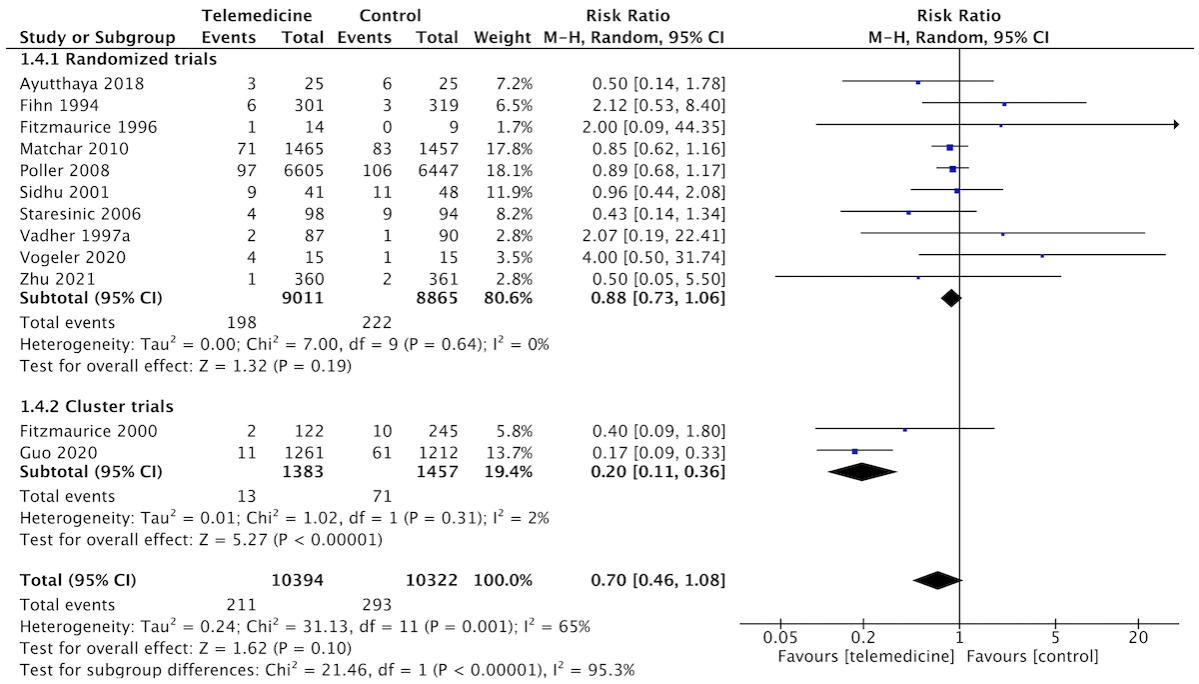
Forest plot of the comparison: telemedicine interventions versus usual care. Outcome: total thromboembolic events.

**Figure 5:**
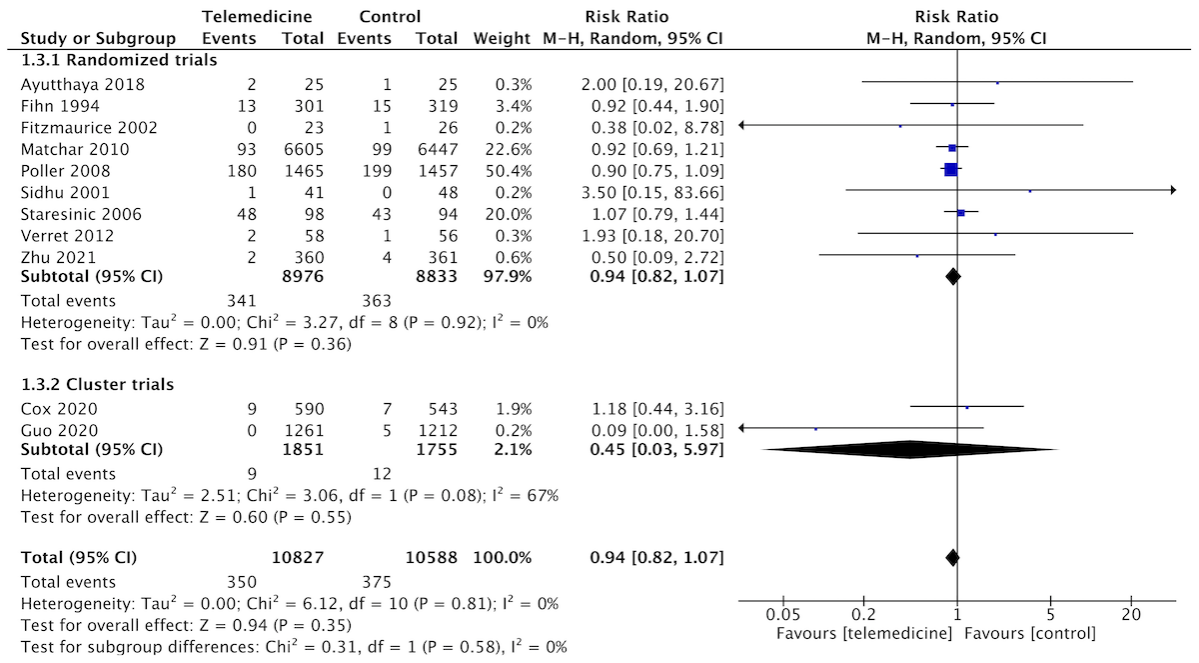
Forest plot of the comparison: telemedicine interventions versus usual care. Outcome: major bleeding.

**Figure 6:**
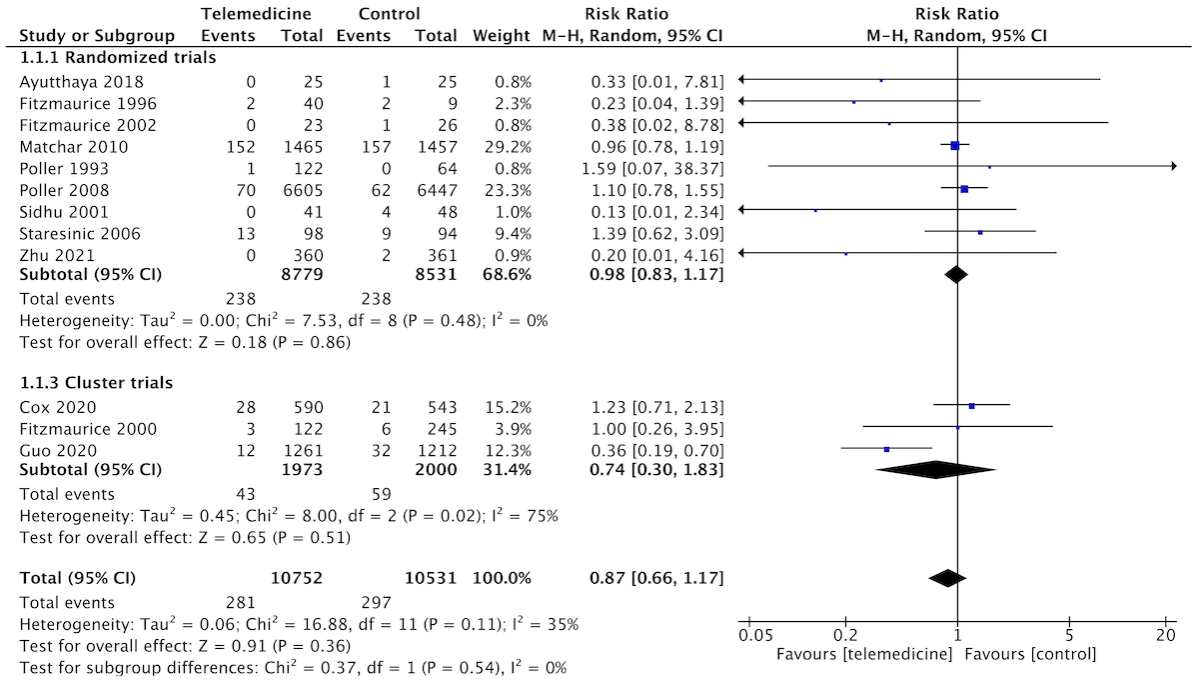
Forest plot of the comparison: telemedicine interventions versus usual care. Outcome: all-cause death.

**Figure 7:**
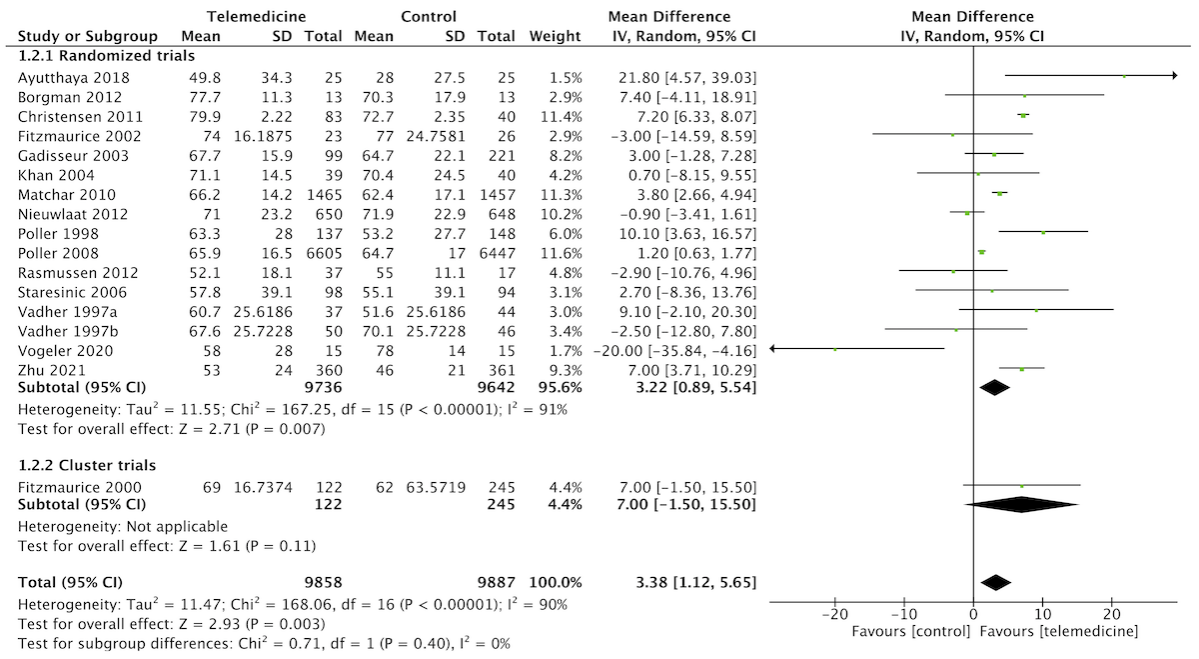
Forest plot of the comparison: telemedicine interventions versus usual care. Outcome: time in therapeutic range.

The main results of our pooled analyses are shown in Table 3, and forest plots are shown in Figures 4-7. Intracluster correlation coefficient values were not obtained for any of the cluster trials included in our meta-analysis. Therefore, we adjusted the results from these trials using an intracluster correlation coefficient of 0.02 before pooling data. This value was reported in similar primary care cluster studies [41] and was used for sample calculation in one of the included trials [40].

Telemedicine resulted in lower rates of TTE compared to usual care (n=13 studies, n=19,223 patients, RR 0.75, 95% CI 0.53-1.07; *I*^2^=42%; Figure 4), although this difference was not statistically significant. The certainty of the evidence was graded as low due to the serious risk of bias in the included studies and imprecision. We decided not to downgrade the certainty for inconsistency, although *I*^2^ suggested moderate heterogeneity because this was entirely explained by the inclusion of 1 trial, as discussed below.

Overall, 11 studies reported rates of major bleeding, and pooled analysis showed that telemedicine is likely to have no impact on that outcome compared to usual care (n=11 studies, n=19,926 patients, RR 0.94, 95% CI 0.82-1.07; *I*^2^=0%; Figure 5). The confidence in that estimate was moderate due to the serious risk of bias in the included studies.

Telemedicine resulted in similar mortality compared to usual care (n=12 studies, n=19,694 patients, RR 0.96, 95% CI 0.78-1.20; *I*^2^= 11%; Figure 6). The certainty of the evidence was graded as moderate due to the serious risk of bias in the included studies.

Moreover, telemedicine resulted in improved TTR compared to usual care (n=16 studies, n=19,609 patients, MD 3.38, 95% CI 1.12-5.65; *I*^2^=90%; Figure 7) though the certainty of the evidence was graded as low due to the serious risk of bias in included studies and inconsistency among studies.

Although the 95% CIs for major bleeding and mortality crossed the null effect, we decided not to downgrade the certainty for imprecision because the intervals were notably narrow, so we considered the true effect to lie in the similarity between both groups.

There was no evidence of publication bias for most evaluated outcomes with a symmetrical distribution of trials across the funnel plots. Mortality was the only outcome with an asymmetrical distribution of studies in the funnel plot with significantly more studies published in favor of intervention. In spite of that asymmetry, Egger’s test resulted in a nonsignificant *P* value (.135). Also, adjusted odds ratio, including the 5 missing studies estimated by Fill and Trim method, indicated that our conclusion would not be significantly altered by a potential publication bias (adjusted odds ratio 0.99, 95% CI 0.83-1.19). Therefore, we decided not to downgrade the confidence in any of the outcomes for publication bias. A detailed analysis of publication bias can be found in Figure S1 and Table S1 in Multimedia Appendix 1.

**Table 3:** Summary of findings: telemedicine compared to usual care for oral anticoagulation management in adult outpatients.

Study design		Studies, n	Certainty assessment					Patients, n/N (%)		Effect		Certainty
			Risk of bias	Inconsistency	Indirectness	Imprecision	Other considerations	Telemedicine	Usual care	Relative (95% CI)	Absolute (95% CI)	
**Total thromboembolic events**												
	Randomized trials	13	Serious^a,b,c^	Not serious^d^	Not serious	Serious^e^	None	204/9657 (2.1)	256/9566 (2.7)	0.75 (0.53-1.07)	7 fewer per 1.000 (from 13 fewer to 2 more)	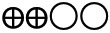 Low
**Major bleeding**												
	Randomized trials	11	Serious^a,b,c^	Not serious	Not serious	Not serious^f^	None	349/10,085 (3.5)	371/9877 (3.8)	0.94 (0.82-1.07)	2 fewer per 1.000 (from 7 fewer to 3 more)	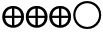 Moderate
**Death**												
	Randomized trials	12	Serious^a,b,c^	Not serious	Not serious	Not serious^f^	None^g^	271/9965 (2.7)	275/9729 (2.8)	0.96 (0.78-1.20)	1 fewer per 1.000 (from 6 fewer to 6 more)	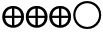 Moderate
**TTR^h^**												
	Randomized trials	16	Serious^a,b,c^	Serious^i^	Not serious	Not serious	None	9813	9796	—^j^	MD^k^ 3.38 higher (1.12 higher to 5.65 higher)	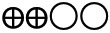 Low

^a^A significant number of trials were not adequately masked. However, this is due to the nature of the intervention, and we judged that it would not significantly impact objective outcomes such as death, thromboembolic and hemorrhagic events, or TTR.

^b^Downgraded for unclear or inadequate randomization process.

^c^Downgraded for high or unclear risk of missing outcome data.

^d^Although *I*^2^ suggested serious heterogeneity, we decided not to downgrade for inconsistency because this is completely explained by the inclusion of 1 study [18].

^e^The CI includes an important benefit but also a small harm, since it slightly crosses the null effect.

^f^We decided not to downgrade for imprecision although 95% CI includes the null effect because the intervals are very narrow and centralized in the null effect, which corroborate similarity between telemedicine and usual care.

^g^Funnel plot shows an asymmetrical distribution of studies, with significantly more studies published in favor of intervention. Egger’s test resulted in a nonsignificant *P* value (.135) and the adjusted odds ratio (OR), including the 5 missing studies estimated by Fill and Trim method, indicated that our conclusion would not be significantly altered by a potential publication bias (OR 0.99, 95% CI 0.83-1.19). Therefore, we decided not to downgrade for publication bias.

^h^TTR: time in therapeutic range.

^i^Despite *I*^2^ of 90%, all but one trial results range from a null effect to a positive effect of telemedicine on TTR. Therefore, we decided to consider it only serious.

^j^Not available.

^k^MD: mean difference.

### Sensitivity and Subgroup Analyses

Sensitivity analyses did not significantly affect the pooled estimated effect for any of the outcomes, neither by the exclusion of each individual study nor by excluding those with a high risk of bias. Likewise, similar pooled effect estimates were obtained when the results of cluster studies were adjusted using an intracluster correlation coefficient of 0.05 (Figure S2 in Multimedia Appendix 1). Nevertheless, excluding Guo’s study from the analysis of TTE reduced the *I*^2^ statistics from 42% to 0%.

Subgroup analyses were carried out for different modalities of telemedicine intervention. Results are shown in Table 4. The only subgroup that yielded a significant result was one of the multitasking interventions, which resulted in a significant reduction of TTE (RR 0.20, 95% CI 0.08-0.48) compared to usual care. Although a better TTR in telemedicine group had already been shown in overall results, the magnitude of the effect in the multitasking application subgroup was larger than in other subgroups.

**Table 4:** Subgroup analysis for different types of telemedicine intervention.

Outcome	Computer-assisted dosing	Laboratory testing + remote adjustment	Self-testing	Multitasking application	*P* value for subgroup differences
Total thromboembolic events, RR^a^ (95% CI)	0.92 (0.71 to 1.20)	0.46 (0.20 to 1.07)	0.90 (0.65 to 1.26)	0.20 (0.08 to 0.48)	.005
Major bleeding, RR (95% CI)	0.90 (0.75 to 1.08)	1.08 (0.80 to 1.45)	0.93 (0.70 to 1.23)	0.84 (0.36 to 1.98)	.77
Death, RR (95% CI)	1.05 (0.76 to 1.45)	1.27 (0.58 to 2.76)	0.84 (0.44 to 1.62)	0.62 (0.20 to 1.92)	.70
TTR^b^, MD^c^ (95% CI)	2.19 (−0.44 to 4.81)	11.06 (−7.51 to 29.63)	3.24 (0.16 to 6.32)	7.00 (3.71 to 10.29)	.12

^a^RR: relative risk.

^b^TTR: time in therapeutic range.

^c^MD: mean difference.

## Discussion

### Principal Findings

This systematic review showed that telemedicine-based OAT management resulted in a better quality of anticoagulation compared to usual care, demonstrated by an improved TTR. The estimated effect for thromboembolic events was not statistically significant. Still, it did show a 25% RR reduction and a 95% CI that barely crossed the null effect, indicating a trend for benefit. In the multitasking intervention subgroup, the reduction in TTE reached a greater magnitude (RR 0.20, 95% CI 0.08-0.48). We also found similar rates of major bleeding and all-cause death in the telemedicine and usual care group. Despite the risk of bias in the included studies, the confidence in those estimates was considered moderate for major bleeding and mortality, as the results were robust and consistent. The confidence level for the other outcomes was low due to the high risk of bias in the included studies as well as imprecision for TTE and inconsistency for TTR.

Three recent systematic reviews [42-44] aimed to answer a similar question, albeit 2 of those focused on telephone-based interventions only. All of them were limited by methodological issues, such as the inclusion of nonrandomized studies, incorrect interpretation of the Cochrane risk of bias tool, classifying studies as having low risk of bias despite having a high or uncertain risk of bias in one of the domains, or a lack of a clear definition of the comparator, including trials in which both treatment and control groups received technology-based interventions [43]. Therefore, an appropriate evidence synthesis, with a comprehensive search, a judicious selection of included studies, and strict methodological criteria, was warranted, and this review meets that evidence gap.

This research was innovative in demonstrating that multitasking telemedicine interventions significantly reduced thromboembolic events and improved anticoagulation quality. This emphasizes the importance of modern telemedicine interventions consisting of bundles of care rather than isolated interventions. Their impact stems from enhanced access to health care, higher quality of care, and better integration of various levels of health services [45]. Technology-based interventions may help implement integrated care of chronic diseases such as AF, heart valve disease, and VTE, beyond anticoagulation management.

Precisely, the Guo et al [18] trial, which tested a multitasking telemedicine intervention for managing patients with AF, found that telemedicine resulted in an important reduction in TTE and mortality. The intervention consisted of a mobile app for integrated management of AF, including anticoagulation indication and management, symptoms control, cardiovascular risk, and comorbidity management, as recommended in current guidelines. The multifaceted intervention, along with the longer follow-up period, may have greatly contributed to the observed effects. The larger impact of the Guo trial, significantly greater than the effect found in any other trial, was probably the reason for the heterogeneity observed in the pooled analysis for TTE, which was abolished after the Guo et al [18] trial exclusion.

The short length of follow-up of most trials may have hindered the impact on clinical outcomes. Nevertheless, it was enough to demonstrate that telemedicine resulted in a better quality of anticoagulation, expressed by an improved TTR. The pooled MD was 3.38 for the entire body of evidence and 7.0 for the multitasking intervention subgroup, highlighting the remarkable impact of multifaceted telemedicine interventions. High heterogeneity in TTR was already anticipated due to the wide range of settings and telehealth strategies in the included studies. Additionally, a higher heterogeneity is usually expected in meta-analyses of continuous outcomes [46]. Different baseline TTRs also could have influenced the impact of the intervention, as it is expected that populations with lower baseline TTRs derive a larger benefit from any intervention that promotes a better quality of therapy [7,34-36]. Even in recent clinical trials of DOAC versus warfarin, TTR in control groups varied widely across various geographical regions [47], reaching values as low as 36% in India. Hence, eHealth implementation may positively impact the quality of anticoagulation, especially in underserved regions.

The complexity and potential hazards associated with OAT, especially VKAs, make it a still underused therapy, and anticipation of difficulty in management is a frequent barrier to an adequate prescription of OAT [48]. Data from different countries and regions show heterogeneous prescription patterns ranging from 76% in high-income countries [49] to as low as 9% in low-income countries [50]. As a result, increasing access to appropriate anticoagulation treatment through telehealth strategies, particularly for underserved populations, may significantly impact their outcomes. This may not be apparent in this research because most studies were conducted in higher-income countries where baseline anticoagulation quality was already high.

Given the rapid uptake of DOAC prescribing worldwide, largely replacing VKAs in many countries, one could question if there will still be a place for telemedicine intervention in managing such treatment in the near future. First of all, VKAs remain the best anticoagulant drug choice in 3 important conditions, that is, antiphospholipid syndrome [51], mechanical heart valves [4], and rheumatic valve disease, as confirmed in a recent trial [5]. Secondly, we included 2 trials addressing DOAC prescribing for patients with AF [18,19]. The telemedicine strategy in both studies incorporated multitasking interventions such as calculating risk scores for thromboembolic and bleeding risks, recommending adjusted DOAC doses based on renal function, age, and other relevant variables, monitoring renal and liver function, suggesting switching from VKA to DOAC when deemed appropriate, and promoting drug adherence through patient diaries and reminders. Therefore, we believe that telemedicine-based OAT management can be beneficial even in the DOAC era, preferably as part of an integrated care pathway.

Concerning costs, evidence is still lacking. In a recently published cost analysis of the ThrombEVAL study [52], the rise in direct costs was outweighed by the lower frequency of adverse events and hospitalizations in patients managed by telemedicine-based intervention, which led to an important reduction in health care expenditures. As cost and reimbursement barriers continue to limit the implementation of telemedicine services, future studies should conduct in-depth cost-effective analyses of the various types of telemedicine strategies to support anticoagulation management. This may help to support public health implementation and the discussion of reimbursement strategies.

This research has some limitations. It included a broad range of different types of telemedicine interventions that may constrain the applicability of our results. However, subgroup analysis should overcome this flaw. The underlying conditions for which anticoagulation was prescribed were also variable, but this reflects the reality of most anticoagulation clinics. Overall, the risk of bias in individual studies was moderate to high. Nonetheless, it is crucial to consider that double-blinding is often impossible due to the nature of the intervention, that is, patients followed remotely by telephone would always know they were allocated to the intervention. Moreover, since we analyzed objective outcomes, the lack of blinding was not considered a major issue. Another limitation was the substantial heterogeneity of TTE and TTR outcomes, as discussed earlier.

### Conclusions

This systematic review provides evidence that telemedicine-based management of OAT results in similar rates of major bleeding and mortality compared to usual care, a trend for a benefit for TTE, and a better quality of anticoagulation, as measured by TTR. Furthermore, telemedicine resulted in an important reduction of TTE in the subgroup of multitasking intervention. Given the potential benefits of telemedicine-based management, such as greater access to remote populations or people with ambulatory restrictions, these findings may encourage further implementation of eHealth strategies for anticoagulation management, particularly as part of multifaceted interventions for integrated care of chronic diseases. Meanwhile, researchers should develop higher-quality evidence focusing on hard clinical outcomes, cost-effectiveness, and quality of life.

## Appendix

Multimedia Appendix 1 Search strategies, full publication bias assessment, cost analysis and sensitivity analysis forest plots.

## Abbreviations

AF: atrial fibrillation
DOAC: direct oral anticoagulant
INR: international normalized ratio
MD: mean difference
OAT: oral anticoagulation therapy
PRISMA: Preferred Reporting Items for Systematic Reviews and Meta-Analyses
RR: relative risk
TTE: thromboembolic event
TTR: time in therapeutic range
VKA: vitamin K antagonist
VTE: venous thromboembolism

## Data Availability

Data will be made available upon request.
