# Supplementary material for: Telemedicine-Based Management of Oral Anticoagulation Therapy: Systematic Review and Meta-analysis
Source: J Med Internet Res. 2023 Jul 10;25:e45922. doi: 10.2196/45922 (PMC10366670; doi:10.2196/45922)
Supplement: Multimedia Appendix 1 [file jmir_v25i1e45922_app1.pdf]

Telemedicine-based oral anticoagulation management: impact on thromboembolic and bleeding events - A systematic review and meta-analysis

Online Supplementary material

Search strategy Medline Pubmed

("Anticoagulants"[Mesh] OR (Anticoagulation Agents) OR (Agents, Anticoagulation) OR (Anticoagulant Agents) OR (Agents, Anticoagulant) OR (Anticoagulant Drugs) OR (Drugs, Anticoagulant) OR (Anticoagulant) OR (Indirect Thrombin Inhibitors) OR (Inhibitors, Indirect Thrombin) OR (Thrombin Inhibitors, Indirect) OR "Warfarin"[Mesh] OR (4-Hydroxy-3-(3-oxo-1-phenylbutyl)-2H-1-benzopyran-2-one) OR (Apo-Warfarin) OR Aldocumar OR (Gen-Warfarin) OR (Warfant) OR Coumadin OR Marevan OR (Warfarin Potassium) OR (Potassium, Warfarin) OR (Warfarin Sodium) OR (Sodium, Warfarin) OR Coumadine OR Tedicumar OR "Dabigatran "[Mesh] OR (N-((2-(((4-(aminoiminomethyl)phenyl)amino)methyl)-1-methyl-1H-benzimidazol-5-yl)carbonyl)-N-2-pyridinyl-beta-alanine) OR (BIBR 1048) OR Pradaxa OR (Dabigatran Etexilate) OR (Etexilate, Dabigatran) OR (Dabigatran Etexilate Mesylate) OR (Etexilate Mesylate, Dabigatran) OR (Mesylate, Dabigatran Etexilate) OR "Rivaroxaban"[Mesh] OR (5-chloro-N-(((5S)-2-oxo-3-(4-(3-oxomorpholin-4-yl)phenyl)-1,3-oxazolidin-5-yl)methyl)thiophene-2-carboxamide) OR Xarelto OR (BAY 59-7939) OR (BAY 59 7939) OR (BAY 597939) "4-Hydroxycoumarins"[Mesh] OR (4 Hydroxycoumarins) OR Phenprocoumon[Mesh] OR (Phenylpropylhydroxycumarinum) OR Phenprocoumalol OR Phenprocoumarol OR Phenprogramma OR Marcoumar OR Marcumar OR Falithrom OR Liquamar OR "Acenocoumarol"[Mesh] OR Nicoumalone OR Acenocoumarin OR Sinthrome OR Synthrom OR Syncoumar OR Syncumar OR Sinkumar OR Sintrom OR (Mini-Sintrom) OR (Mini Sintrom) OR (MiniSintrom) OR "Dicumarol"[Mesh] OR Bishydroxycoumarin OR Dicoumarol OR Dicoumarin OR "Factor Xa Inhibitors"[Mesh] OR (Direct Factor Xa Inhibitors) OR Apixaban OR Edoxaban OR Betrixaban)

AND

("Telemedicine"[Mesh] OR (mobile health) OR (health, mobile) OR (health) OR (telehealth) OR (ehealth) OR Telenursing[Mesh] OR tele cardiology OR tele neurology OR "Medical Informatics"[Mesh] OR (health informatics) OR (informatics, health) OR (informatics, medical) OR (clinical informatics) OR (informatics, clinical) OR (medical computer science) OR (computer science, medical) OR (medical computer sciences) OR (science, medical computer) OR (health information technology) OR (health information technologies) OR (information technology, health) OR (technology, health information) OR (information science, medical) OR (medical information sciences) OR (medical information science) OR "Information Systems"[Mesh] OR (information system) OR (system, information) OR (systems, information) OR (information retrieval systems) OR (information retrieval system) OR (system, information retrieval) OR (systems, information retrieval) OR (ancillary information systems) OR (ancillary information systems) OR (information system, ancillary) OR (information systems, ancillary) OR (system, ancillary information) OR (systems, ancillary information) OR (emergency care information systems) OR "Remote Consultation"[Mesh] OR (consultation, remote) OR teleconsultation OR teleconsultations OR "Telephone"[Mesh] OR (telephones) OR (switchboard service) OR (service, switchboard) OR (services, switchboard) OR (switchboard services) OR "Videoconferencing"[Mesh] OR (videoconferencing) OR (videoconference) OR (videoconferences) OR telediagnosis OR telediagnosis OR "Decision Making, Computer-Assisted"[Mesh] OR (decision making, computer

assisted) OR (computer-assisted decision making) OR (computer assisted decision making) OR (medical decision making, computer-assisted) OR (medical decision making, computer assisted) OR "Clinical Decision Making"[Mesh] OR (clinical decision making) OR (decision-making, clinical) OR (medical decision-making) OR (decision-making, medical) OR (medical decision making) OR (tele cardiology) OR (tele neurology) OR "digital health")

Search strategy EMBASE

(‘Telemedicine’/exp OR ‘tele medicine’ OR ‘telenursing’/exp OR ‘tele-nursing’ OR 'telecardiology'/exp OR 'tele-cardiology' OR ‘Medical informatics’/exp OR ‘health informatics’ OR ‘health information technology’ OR ‘medical data processing’ OR ‘medical informatics applications’ OR ‘medical informatics computing’ OR ‘medical information technology’ OR ‘public health informatics’ OR ‘Health information systems’/exp OR ‘clinical information system’ OR ‘clinical pharmacy information systems’ OR ‘health information exchange’ OR ‘health information management’ OR ‘health information manager’ OR ‘health information network’ OR ‘health information system’ OR ‘health information systems’ OR ‘IS-H med’ OR ‘medical information service’ OR ‘Information System’/exp OR ‘information management’ OR ‘information systems’ OR ‘integrated advanced information management systems’ OR ‘management information system’ OR ‘management information systems’ OR ‘management, information’ OR ‘personnel staffing and scheduling information systems’ OR ‘teleconsultation’/exp OR ‘remote consultation’ OR ‘tele-consultation’ OR ‘telephone consultation’ OR ‘mobile phone’/exp OR ‘cell phone’ OR ‘cell phones’ OR ‘cellphone’ OR ‘cellphones’ OR ‘cellular phone’ OR ‘cellular telephone’ OR ‘mobile telephone’ OR ‘Telephone’/exp OR ‘dataphone’ OR ‘telephone line’ OR ‘clinical decision support system’/exp OR ‘CDS system (clinical decision support system)’ OR ‘clinical decision support (CDS) system’ OR ‘clinician decision support system’ OR ‘decision support systems’ OR ‘clinical’ ‘videoconferencing’/exp OR ‘video conference’ OR ‘video conferencing’ OR ‘videoconference’ OR ‘decision support system’/exp OR ‘decision making, computer-assisted’ OR ‘decision support’ OR ‘decision support systems, management’ OR ‘decision support techniques’ OR ‘clinical decision making’/exp OR ‘clinical decision-making’ OR ‘Reminder System’/exp OR ‘reminder systems’ OR ‘Telecardiology’/exp OR ‘tele-cardiology’ OR ‘Tele neurology’ OR ‘Information and communication technologies’)
AND
(‘anticoagulant agent’/exp OR ‘anti coagulant’ OR ‘anti coagulant agent’ OR ‘anti coagulant drug’ OR ‘anti coagulating agent’ OR ‘anticoagulant’ OR ‘anticoagulant drug’ OR ‘anticoagulants’ OR ‘anticoagulating agent’ OR ‘anticoagulation agent’ OR ‘anticoagulative agent’ OR ‘antithrombotic’ OR ‘antithrombotic agent’ OR ‘antithrombotic drug’ OR ‘oral anticoagulant’ OR ‘oral anticoagulant agent’ OR ‘Warfarin’/exp OR ‘1 (4 hydroxy 3 coumarinyl) 1 phenyl 3 butanone’ OR ‘3 (alpha acetonylbenzyl) 4 hydroxycoumarin’ OR ‘3 acetonylbenzonyl 4 hydroxy coumarinedimethylaminoethanol’ OR ‘3 alpha phenyl beta acetylethyl 4 hydroxycoumarin’ OR ‘3 (alpha acetonylbenzyl) 4 hydroxycoumarin’ OR ‘acetonylbenzylhydroxycoumarin’ OR ‘adoisine’ OR ‘aldocumar’ OR ‘alpha acetonylbenzyl 4 hydroxycoumarin dimethylaminoethanol’ OR ‘antrombin k’ OR ‘athrombin’ OR ‘athrombin k’ OR ‘athrombin-k’ OR ‘athrombine k’ OR ‘athrombinek’ OR ‘befarin’ OR ‘carfin’ OR ‘circuvit’ OR ‘compound 42’ OR ‘coumadan’ OR ‘coumadan sodico’ OR ‘coumadin’ OR ‘coumadin sodium’ OR ‘coumadine’ OR ‘coumafene’ OR ‘coumaphene’ OR ‘d warfarin’ OR ‘dagonal’ OR ‘dextro

warfarin’ OR ‘farin’ OR ‘jantoven’ OR ‘kumatox’ OR ‘l warfarin’ OR ‘levo warfarin’ OR ‘maforan’ OR ‘marevan’ OR ‘orfarin’ OR ‘panwarfarin’ OR ‘panwarfin’ OR ‘potassium warfarin’ OR ‘prothromadin’ OR ‘r warfarin’ OR ‘simarc-2’ OR ‘sodium warfarin’ OR ‘sodium warfarinum’ OR ‘sofarin’ OR ‘tintorane’ OR ‘uniwarfin’ OR ‘wafarin’ OR ‘waran’ OR ‘warf compound 42’ OR ‘warfar’ OR ‘warfarin 2 (dimethylamino) ethanol’ OR ‘warfarin potassium’ OR ‘warfarin sodium’ OR ‘warfarine’ OR ‘warfarinum sodium’ OR ‘warfil 5’ OR ‘warfilone’ OR ‘warnerin’ OR ‘Dabigatran‘/exp OR ‘bibr 953; bibr953’ OR ‘n [[2 [(4 amidinoanilino) methyl] 1 methyl 5 benzimidazolyl] carbonyl] n (2 pyridyl) beta alanine’ OR ‘n [[2 [[[4 (aminoiminomethyl) phenyl] amino] methyl] 1 methyl 1h benzimidazol 5 yl] carbonyl] n (2 pyridyl) beta alanine’ OR ‘Rivaroxaban’/exp OR ‘5 chloro n [[2 oxo 3 [4 (3 oxomorpholin 4 yl) phenyl] 1, 3 oxazolidin 5 yl] methyl] thiophene 2 carboxamide’ OR ‘5 chloro n [[2 oxo 3 [4 (3 oxomorpholin 4 yl) phenyl] oxazolidin 5 yl] methyl] thiophene 2 carboxamide’ OR ‘5 chloro n [[2 oxo 3 [4 (3 oxomorpholino) phenyl] 5 oxazolidinyl] methyl] 2 thiophenecarboxamide’ OR ‘bay 59 7939’ OR ‘bay 59-7939’ OR ‘bay 597939’ OR ‘bay59 7939’ OR ‘bay59-7939’ OR ‘bay597939’ OR ‘xarelto’ OR ‘4 hydroxycoumarin derivative’/exp OR ‘4 hydroxycoumarins’ OR ‘4-hydroxycoumarins’ OR 'phenprocoumon'/exp OR '3 (1 phenylpropyl) 4 hydroxycoumarin' OR '3 (alpha ethylbenzyl) 4 hydroxycoumarin' OR '4 hydroxy 3 (1 phenylpropyl) coumarin' OR 'falithrom' OR 'falithrome' OR 'fenprocoumon' OR 'liquamar' OR 'marcoumar' OR 'marcumar' OR 'phenprocouman' OR 'phenprocoumarol' OR 'phenprocoumom' OR 'phenprocumarol' OR 'phenylpropylhydroxycoumarin' OR 'phenylpropyloxycoumarin' OR ‘Acenocoumarol’/exp OR ‘3 (alpha acetonyl 4 nitrobenzyl) 4 hydroxycoumarin’ OR ‘3 (alpha acetonyl para nitrobenzyl) 4 hydroxycoumarin’ OR ‘3 (alpha para nitrophenyl beta acetylethyl) 4 hydroxycoumarin’ OR ‘3 [2 acetyl 1 (para nitrophenyl) ethyl] 4 hydroxycoumarin’ OR ‘3 [alpha (4 nitrophenyl) beta acetylethyl] 4 hydroxycoumarin’ OR ‘3alpha (4 nitrophenyl) beta (acetylethyl) 4 oxycoumarine’ OR ‘acenocoumarin’ OR ‘acenocoumarine’ OR ‘acenocoumarole’ OR ‘acenocoumarolum’ OR ‘acenocumarol’ OR ‘acenocumarolo’ OR ‘acenocumerol’ OR ‘acenokumarin’ OR ‘acitrom’ OR ‘ascumar’ OR ‘coumarin g 23350’ OR ‘g 23, 350’ OR ‘g 23350’ OR ‘g-23, 350’ OR ‘g-23350’ OR ‘g23, 350’ OR ‘g2335’ OR ‘g23350’ OR ‘neo sintrom’ OR ‘neo-sintrom’ OR ‘neosintrom’ OR ‘neositron’ OR ‘nicoumalone’ OR ‘nicumalon’ OR ‘niffcoumar’ OR ‘nitrovarfarian’ OR ‘nitrowarfarin’ OR ‘sincoumar’ OR ‘sincumar’ OR ‘sinkumar’ OR ‘sinthrom’ OR ‘sinthrome’ OR ‘sintrom’ OR ‘sintroma’ OR ‘sintron’ OR ‘syncoumar’ OR ‘syncumar’ OR ‘syntrom’ OR ‘trombostop’ OR ‘zotil’ OR ‘Dicumarol’/exp OR ‘3, 3 methylenebis (4 hydroxy 1, 2 benzopyrone)’ OR ‘3, 3 methylenebis (4 hydroxycoumarin)’ OR ‘bis hydroxycoumarin’ OR ‘biscumarolum’ OR ‘bishydroxy coumarin’ OR ‘bishydroxycoumarin’ OR ‘bishydroxycumarin’ OR ‘cumaphos’ OR ‘cumarene’ OR ‘cumid’ OR ‘dicoumarin’ OR ‘dicoumarine’ OR ‘dicuman’ OR ‘dicumarol’ OR ‘dicumol’ OR ‘discoumarol’ OR ‘dufalone’ OR ‘embolin’ OR ‘kumoran’ OR ‘melitoxin’ OR ‘temparin’ OR ‘blood clotting factor 10a inhibitor’/exp OR ‘blood clotting factor xa inhibitor’ OR ‘direct factor Xa inhibitor’ OR ‘direct factor Xa inhibitors’ OR ‘factor Xa inhibitor’ OR ‘factor Xa inhibitors’ OR ‘apixaban’/exp OR ‘1 (4 methoxyphenyl) 7 oxo 6 [4 (2 oxopiperidin 1 yl) phenyl] 4, 5, 6, 7 tetrahydro 1h pyrazolo [3, 4 c] pyridine 3 carboxamide’ OR ‘4, 5, 6, 7 tetrahydro 1 (4 methoxyphenyl) 7 oxo 6 [4 (2 oxo 1 piperidinyl) phenyl] 1h pyrazolo [3, 4 c] pyridine 3 carboxamide’ OR ‘bms 562247’ OR ‘bms 562247 01’ OR ‘bms 562247-01’ OR ‘bms562247’ OR ‘bms562247 01’ OR ‘bms562247-01’ OR ‘eliques’ OR ‘eliquis’ OR ‘Edoxaban’ OR ‘du 176’ OR ‘du 176b’ OR ‘du176’ OR ‘du176b’ OR ‘edoxaban tosilate’ OR ‘edoxaban tosilate hydrate’ OR

‘edoxaban tosylate’ OR ‘edoxaban tosylate hydrate’ OR ‘endoxaban’ OR ‘lixiana’ OR ‘n (5 chloro 2 pyridinyl) n [4 (n, n dimethylcarbamoyl) 2 (5 methyl 4, 5, 6, 7 tetrahydrothiazolo [5, 4 c] pyridine 2 carboxamido) cyclohexyl] oxamide’ OR ‘roteas’ OR ‘savaysa’ OR ‘Betrixaban’/exp OR ‘bevyxxa’ OR ‘dexxience’ OR ‘n (5 chloro 2 pyridinyl) 2 [4 (n, n dimethylcarbamimidoyl) benzamido] 5 methoxybenzamide’ OR ‘n (5 chloropyridin 2 yl) 2 [[4 (n, n dimethylcarbamimidoyl) benzoyl] amino] 5 methoxybenzamide’ OR ‘prt 054021’ OR ‘prt054021’)

Search strategy Cochrane

ID	Search	Hits
#1	MeSH descriptor: [Telenursing] explode all trees	30
#2	MeSH descriptor: [Medical Informatics] explode all trees	9757
#3	MeSH descriptor: [Medical Informatics Applications] explode all trees	9696
#4	MeSH descriptor: [Health Information Systems] explode all trees	11
#5	MeSH descriptor: [Information Systems] explode all trees	2256
#6	MeSH descriptor: [Remote Consultation] explode all trees	374
#7	MeSH descriptor: [Decision Support Systems, Clinical] explode all trees	350
#8	MeSH descriptor: [Electronic Health Records] explode all trees	337
#9	MeSH descriptor: [Artificial Intelligence] explode all trees	994
#10	MeSH descriptor: [Patient Portals] explode all trees	8
#11	MeSH descriptor: [Videoconferencing] explode all trees	187
#12	MeSH descriptor: [Decision Making, Computer-Assisted] explode all trees	5173
#13	MeSH descriptor: [Clinical Decision-Making] explode all trees	185
#14	MeSH descriptor: [Reminder Systems] explode all trees	893
#15	MeSH descriptor: [Anticoagulants] explode all trees	4474
#16	MeSH descriptor: [Warfarin] explode all trees	1581
#17	MeSH descriptor: [Dabigatran] explode all trees	266
#18	MeSH descriptor: [Rivaroxaban] explode all trees	423
#19	MeSH descriptor: [4-Hydroxycoumarins] explode all trees	1788
#20	MeSH descriptor: [Phenprocoumon] explode all trees	105
#21	MeSH descriptor: [Acenocoumarol] explode all trees	129
#22	MeSH descriptor: [Dicumarol] explode all trees	22
#23	MeSH descriptor: [Factor Xa Inhibitors] explode all trees	502
#24	Apixaban	910
#25	Edoxaban	577
#26	Betrixaban	118
#27	Telecardiology	34
#28	Tele cardiology	45
#29	Information and communication technologies	564
#30	Tele neurology	26
#31	Teleneurology	2
#33	#1 OR #2 OR #3 OR #4 OR #5	9786

#34 #6 OR #7 OR #8 OR #9 OR #10 2877
#35 #11 OR #12 OR #13 OR #14
#36 #15 OR #16 OR #17 OR #18 OR #19 OR #20
#37 #21 OR #22 OR #23 OR #24 OR #25 OR #26
#38 #33 OR #34 OR #35 OR #27 OR #28 OR #29 OR #30 OR 31
#39 #36 OR #37
#40 #38 AND #39

Search strategy LILACS
(("Telemedicine") OR ("Telemedicina") OR ("Telemedicina") OR ("Connected Health") OR ("Digital Health") OR ("Health 2.0") OR ("Health Tele-Services") OR ("Health Teleservices") OR ("Health, Mobile") OR ("Medicine 2.0") OR ("Mobile Health") OR ("Pervasive Computing Technologies for Healthcare") OR ("Pervasive Health") OR ("Telecare") OR ("Telecure") OR ("Telehealth") OR ("Teleservices in the Health Sector") OR ("Ubiquitous Health") OR ("eHealth") OR ("mHealth") OR ("mHealth Alliance") OR ("u-Health") OR ("Telenursing") OR ("Teleenfermería") OR ("Telenfermagem") OR ("Medical Informatics") OR ("Informática Médica") OR ("Informática Médica") OR ("Clinical Informatics") OR ("Computer Science, Medical") OR ("Health Informatics") OR ("Health Information Technologies") OR ("Health Information Technology") OR ("Informatics, Clinical") OR ("Informatics, Health") OR ("Informatics, Medical") OR ("Information Science, Medical") OR ("Information Technology, Health") OR ("Medical Computer Science") OR ("Medical Computer Sciences") OR ("Medical Information Science") OR ("Medical Information Sciences") OR ("Science, Medical Computer") OR ("Technology, Health Information") OR ("Information Systems") OR ("Sistemas de Información") OR ("Sistemas de Informação") OR ("Ancillary Information System") OR ("Ancillary Information Systems") OR ("Emergency Care Information Systems") OR ("Information Retrieval System") OR ("Information Retrieval Systems") OR ("Information System") OR ("Information System, Ancillary") OR ("Information Systems, Ancillary") OR ("System, Ancillary Information") OR ("System, Information") OR ("System, Information Retrieval") OR ("Systems, Ancillary Information") OR ("Systems, Information") OR ("Systems, Information Retrieval") OR ("Remote Consultation") OR ("Consulta Remota") OR ("Consulta Remota") OR ("Asynchronous Teleconsultation") OR ("Consultation, Remote") OR ("Synchronous Teleconsultation") OR ("Teleconsultation") OR ("Teleconsultations") OR ("Telephone") OR ("Teléfono") OR ("Telefone") OR ("Fixed-Line") OR ("Home Phone") OR ("Land Line") OR("Land-Line") OR ("Landline") OR ("Service, Switchboard") OR ("Services, Switchboard") OR ("Switchboard Service") OR ("Switchboard Services") OR ("Telephone Call") OR ("Telephone Calls") OR ("Telephones") OR ("Wireline") OR ("Videoconferencing") OR ("Videoconferencia") OR ("Videoconferência") OR ("Videoconference") OR ("Videoconferences") OR ("Videoconferencings") OR ("Decision Making, Computer-Assisted") OR ("Toma de Decisiones Asistida por Computador") OR ("Tomada de Decisões Assistida por Computador") OR ("Computer Assisted Decision Making") OR ("Computer-Assisted Decision Making") OR ("Decision Making, Computer Assisted") OR ("Medical Decision Making, Computer Assisted") OR ("Medical Decision Making, Computer-Assisted") OR ("Clinical Decision-Making") OR ("Toma de Decisiones Clínicas") OR ("Tomada de Decisão Clínica") OR ("Clinical Decision Making") OR ("Decision-Making, Clinical") OR ("Decision-Making, Medical") OR ("Medical

Decision Making") OR ("Medical Decision-Making"))

AND

(("Anticoagulants") OR ("Anticoagulantes") OR ("Anticoagulantes") OR ("Agents, Anticoagulant") OR ("Agents, Anticoagulation") OR ("Anticoagulant") OR ("Anticoagulant Agents") OR ("Anticoagulant Drugs") OR ("Anticoagulation Agents") OR ("Drugs, Anticoagulant") OR ("Indirect Thrombin Inhibitors") OR ("Inhibitors, Indirect Thrombin") OR ("Thrombin Inhibitors, Indirect") OR ("Warfarin") OR ("Warfarina") OR ("Varfarina") OR ("4-Hydroxy-3-(3-oxo-1-phenylbutyl)-2H-1-benzopyran-2-one") OR ("Aldocumar") OR ("Apo-Warfarin") OR ("Coumadin") OR ("Coumadine") OR ("Gen-Warfarin") OR ("Marevan") OR ("Potassium, Warfarin") OR ("Sodium, Warfarin") OR ("Tedicumar") OR ("Warfant") OR ("Warfarin Potassium") OR ("Warfarin Sodium") OR ("Dabigatran") OR ("Dabigatrán") OR ("Dabigatrana") OR ("BIBR 1048") OR ("Dabigatran Etexilate") OR ("Dabigatran Etexilate Mesylate") OR ("Etexilate Mesylate, Dabigatran") OR ("Etexilate, Dabigatran") OR ("Mesylate, Dabigatran Etexilate") OR ("N-((2-(((4-(aminoiminomethyl)phenyl)amino)methyl)-1-methyl-1H-benzimidazol-5-yl)carbonyl)-N-2-pyridinyl-beta-alanine") OR ("Pradaxa") OR ("Rivaroxaban") OR ("Rivaroxabán") OR ("Rivaroxabana") OR ("5-chloro-N-(((5S)-2-oxo-3-(4-(3-oxomorpholin-4-yl)phenyl)-1,3-oxazolidin-5-yl)methyl)thiophene-2-carboxamide") OR ("BAY 59 7939") OR ("BAY 59-7939") OR ("BAY 597939") OR ("Xarelto ") OR ("4-Hydroxycoumarins") OR ("4-Hidroxicumarinas") OR ("4-Hidroxicumarinas") OR ("4 Hydroxycoumarins") OR ("Phenprocoumon") OR ("Fenprocumón") OR ("Femprocumona") OR ("Falithrom") OR ("Liquamar") OR ("Marcoumar") OR ("Marcumar") OR ("Phenprocoumalol") OR ("Phenprocoumarol") OR ("Phenprogramma") OR ("Phenylpropylhydroxycumarinum") OR ("Acenocoumarol") OR ("Acenocumarol") OR ("Acenocumarol") OR ("Acenocoumarin") OR ("Mini Sintrom") OR ("Mini-Sintrom") OR ("MiniSintrom") OR ("Nicoumalone") OR ("Sinkumar") OR ("Sinthrome") OR ("Sintrom") OR ("Syncoumar") OR ("Syncumar") OR ("Synthrom") OR ("Dicumarol") OR ("Dicumarol") OR ("Dicumarol") OR ("Bishydroxycoumarin") OR ("Dicoumarin") OR ("Dicoumarol") OR ("Factor Xa Inhibitors") OR ("Inhibidores del Factor Xa") OR ("Inibidores do Fator Xa") OR ("Direct Factor Xa Inhibitors"))

Seach strategy Google Scholar

Anticoagulation

AND

(telemedicine OR "health information system" OR "Decision support system" OR "Remote Consultation" OR "Medical informatics" OR telehealth)

Costs

Only five of the studies included in this systematic review mentioned cost comparisons between telemedicine and control groups. Matchar et al[24] found that telemedicine intervention associated with self-testing resulted in higher costs, although not significantly different from those in the usual care group (difference $ 1,249; 95% CI, -1,205 to 3,703, P=0.32). An economic analysis by Fitzmaurice [17] also detected a higher cost in the telemedicine group, which costs on average approximately $160 per patient per year more than usual care. The excess costs mainly resulted from capital investment in equipping clinics and the higher frequency of testing found within the telemedicine practices. Fitzmaurice (1996)[27] showed a higher cost in the telemedicine group due to the purchase of the software, which was offset by the savings that resulted from the reduction in outpatient hospital visits. In Fitzmaurice's 2002 trial[9], the mean cost per patient-year in the intervention arm was £425 compared with £90 for patients in the control arm ($p < 0.001$). Intervention costs were based on capital costs and running costs of the equipment, quality control, training, and support from the practice. A recently published cost analysis of the ThrombEVAL study[52] also showed that direct costs were increased in the telemedicine arm (€311 per patient-year versus €101 per patient-year in usual care) but the intervention resulted in a reduction of hospitalizations, which led to an important reduction in healthcare expenditures. Hospitalization costs were €3,261 per patient-year in the usual care group and €683 per patient-year in the Telemedicine group.

Figure S1: Funnel plots for different outcomes

A. **Total thromboembolic events**

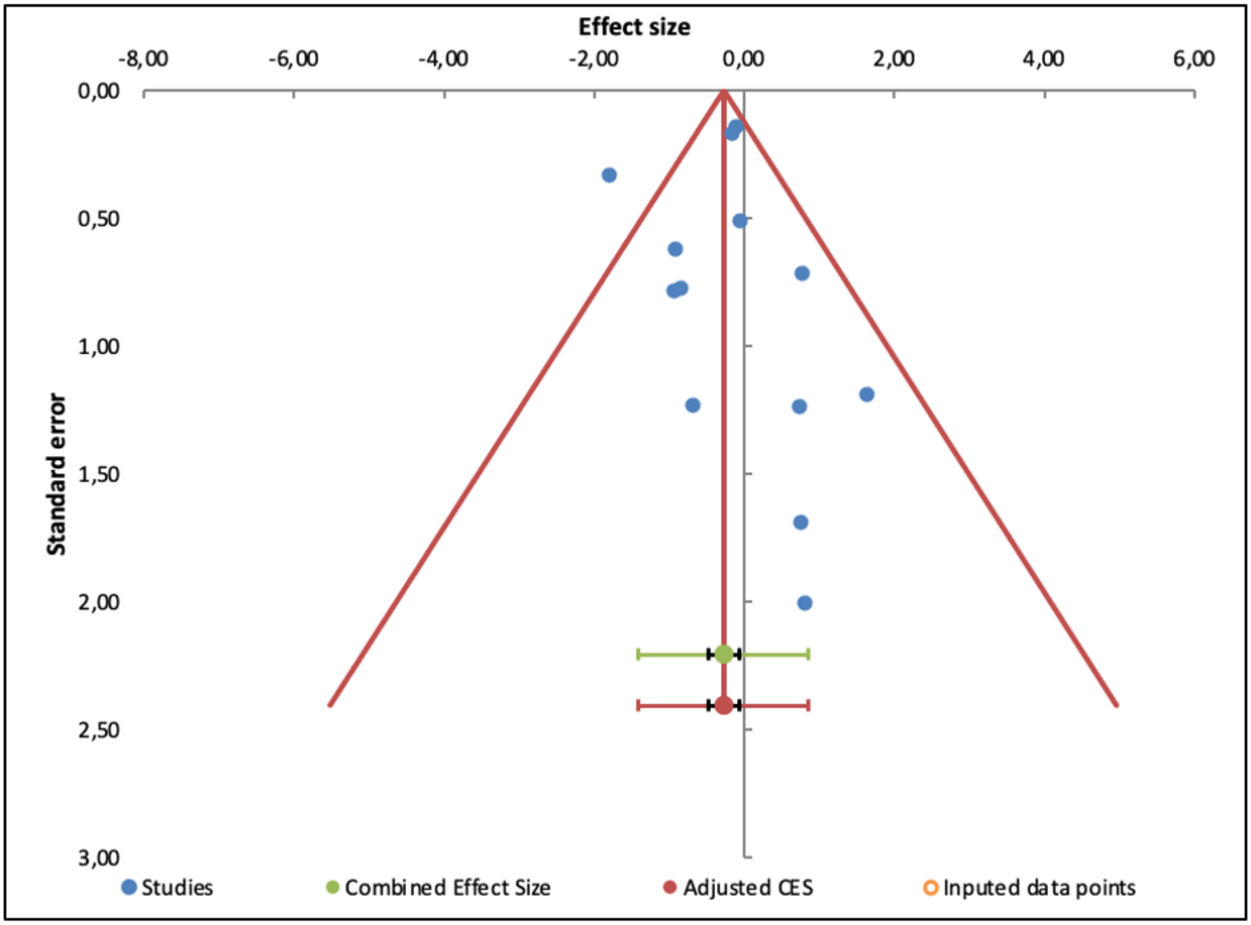

B. **Major Bleeding**

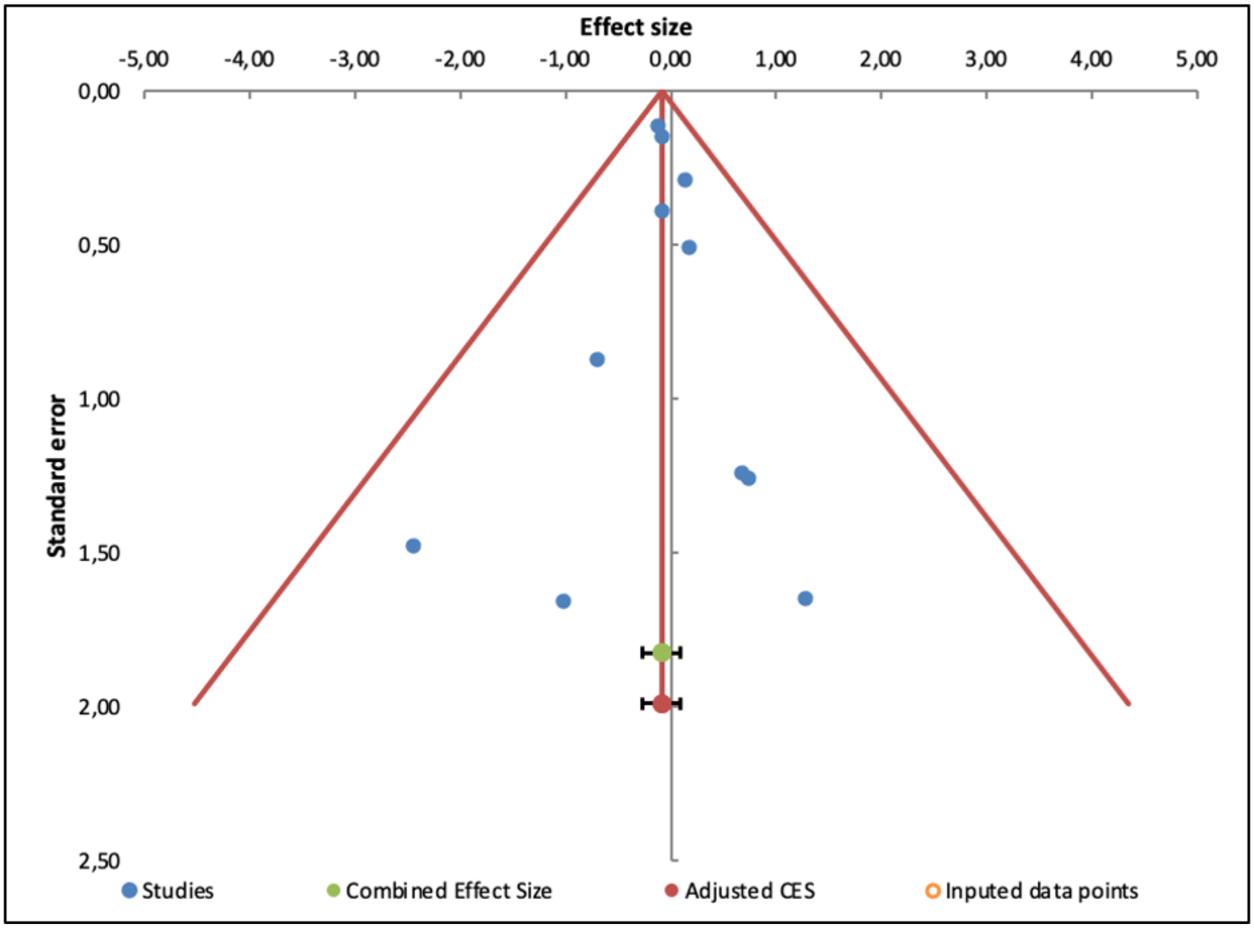

C. All-cause death

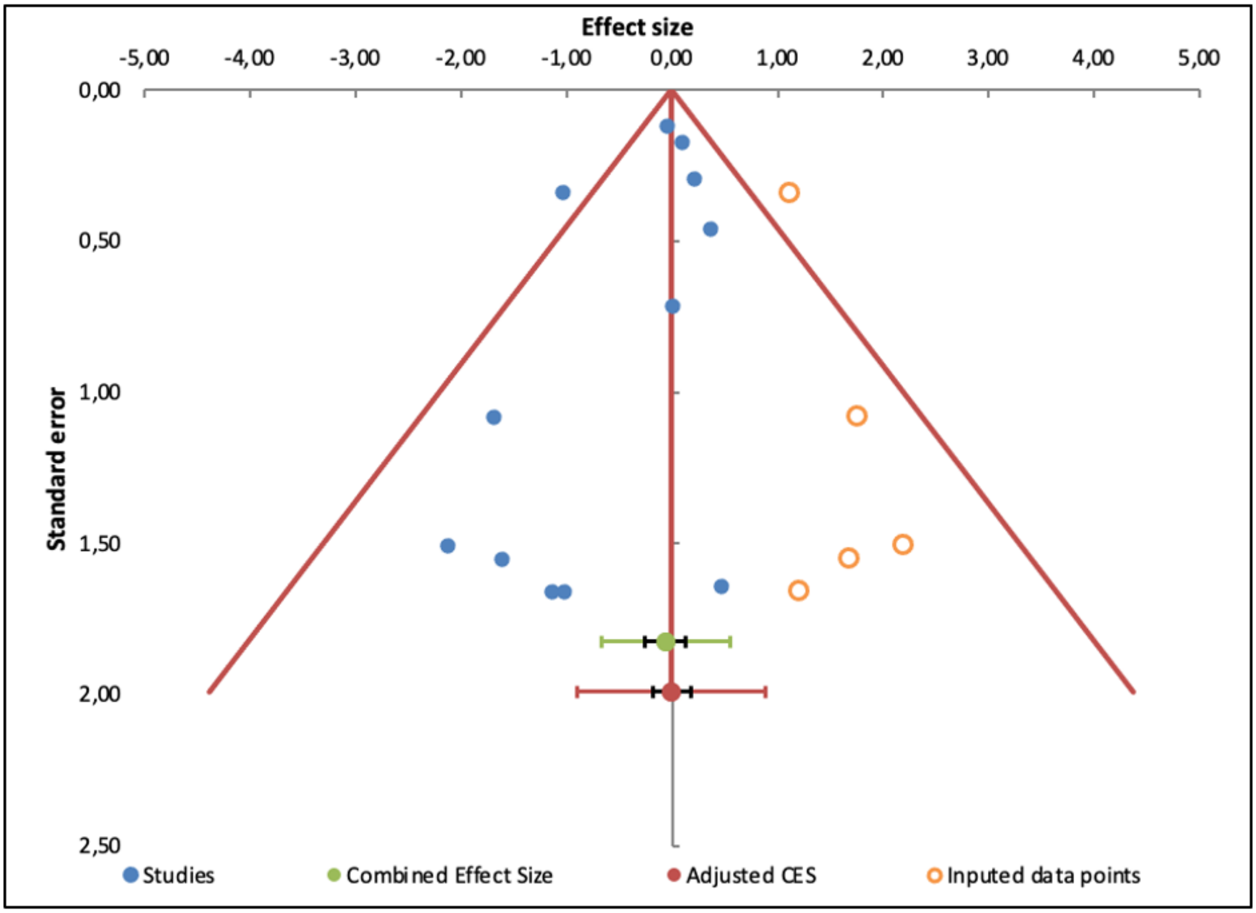

D. Time in therapeutic range (TTR)

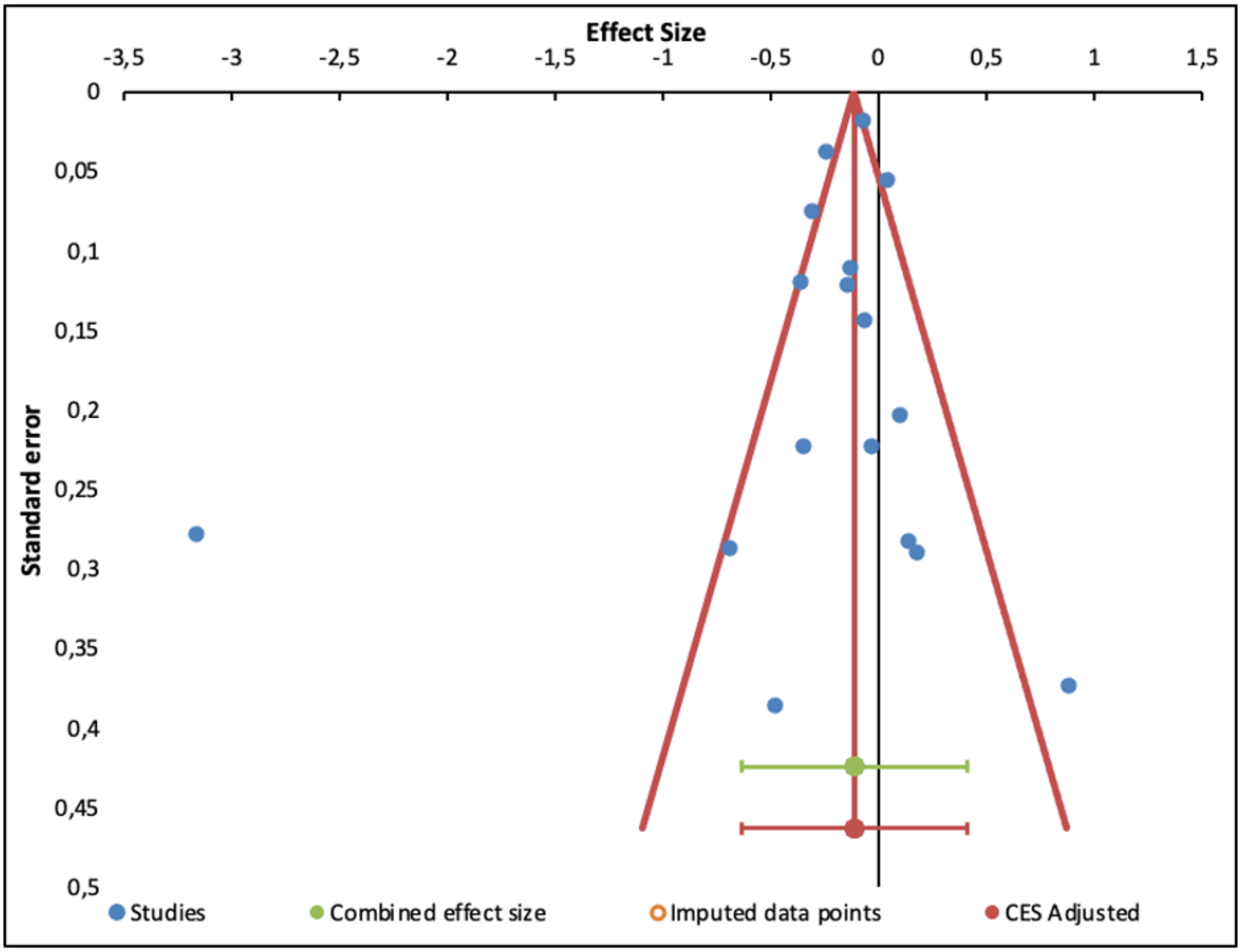

Table S1: Publication Bias Analysis

	**Egger Regression - p value**	**Trim and Fill - number of missing studies**
**Total Thromboembolic Events**	0.965	0
**Major Bleeding**	0.928	0
**All-cause death**	0.135	5
**Time in therapeutic range**	0.251	0

A. Total thromboembolic events

Study or Subgroup	Telemedicine Events	Telemedicine Total	Control Events	Control Total	Weight	Risk Ratio M-H, Random, 95% CI
**1.13.1 Randomized trials**						
Ayutthaya 2018	3	25	6	25	3.8%	0.50 [0.14, 1.78]
Fihn 1994	6	301	3	319	3.3%	2.12 [0.53, 8.40]
Fitzmaurice 1996	1	14	0	9	0.7%	2.00 [0.09, 44.35]
Matchar 2010	71	1465	83	1457	32.7%	0.85 [0.62, 1.16]
Poller 2008	97	6605	106	6447	36.4%	0.89 [0.68, 1.17]
Sidhu 2001	9	41	11	48	9.3%	0.96 [0.44, 2.08]
Staresinic 2006	4	98	9	94	4.6%	0.43 [0.14, 1.34]
Vadher 1997a	2	87	1	90	1.1%	2.07 [0.19, 22.41]
Vogeler 2020	4	15	1	15	1.5%	4.00 [0.50, 31.74]
Zhu 2021	1	360	2	361	1.1%	0.50 [0.05, 5.50]
Subtotal (95% CI)		9011		8865	94.6%	0.88 [0.73, 1.06]
Total events	198		222			
Heterogeneity: $Tau^2 = 0.00$; $Chi^2 = 7.00$, df = 9 (P = 0.64); $I^2 = 0\%$						
Test for overall effect: Z = 1.32 (P = 0.19)						
**1.13.2 Cluster trials**						
Fitzmaurice 2000	1	49	4	99	1.4%	0.51 [0.06, 4.40]
Guo 2020	3	312	15	300	4.1%	0.19 [0.06, 0.66]
Subtotal (95% CI)		361		399	5.4%	0.24 [0.08, 0.71]
Total events	4		19			
Heterogeneity: $Tau^2 = 0.00$; $Chi^2 = 0.58$, df = 1 (P = 0.45); $I^2 = 0\%$						
Test for overall effect: Z = 2.59 (P = 0.010)						
**Total (95% CI)**		9372		9264	100.0%	0.83 [0.64, 1.07]
Total events	202		241			
Heterogeneity: $Tau^2 = 0.03$; $Chi^2 = 12.99$, df = 11 (P = 0.29); $I^2 = 15\%$						
Test for overall effect: Z = 1.45 (P = 0.15)						
Test for subgroup differences: $Chi^2 = 5.40$, df = 1 (P = 0.02), $I^2 = 81.5\%$						

Risk Ratio
M-H, Random, 95% CI

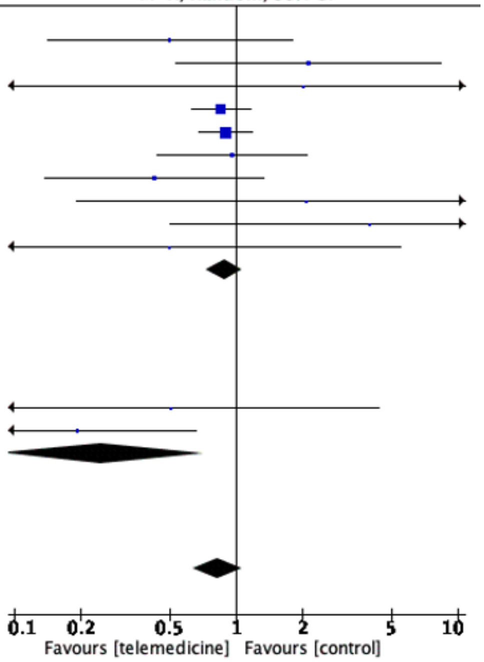

B. Major bleeding

Study or Subgroup	Telemedicine Events	Telemedicine Total	Control Events	Control Total	Weight	Risk Ratio M-H, Random, 95% CI
**1.12.1 Randomized trials**						
Ayutthaya 2018	2	25	1	25	0.3%	2.00 [0.19, 20.67]
Fihn 1994	13	301	15	319	3.4%	0.92 [0.44, 1.90]
Fitzmaurice 2002	0	23	1	26	0.2%	0.38 [0.02, 8.78]
Matchar 2010	93	6605	99	6447	22.7%	0.92 [0.69, 1.21]
Poller 2008	180	1465	199	1457	50.6%	0.90 [0.75, 1.09]
Sidhu 2001	1	41	0	48	0.2%	3.50 [0.15, 83.66]
Staresinic 2006	48	98	43	94	20.0%	1.07 [0.79, 1.44]
Verret 2012	2	58	1	56	0.3%	1.93 [0.18, 20.70]
Zhu 2021	2	360	4	361	0.6%	0.50 [0.09, 2.72]
Subtotal (95% CI)		8976		8833	98.3%	0.94 [0.82, 1.07]
Total events	341		363			
Heterogeneity: $Tau^2 = 0.00$; $Chi^2 = 3.27$, df = 8 (P = 0.92); $I^2 = 0\%$						
Test for overall effect: Z = 0.91 (P = 0.36)						
**1.12.2 Cluster trials**						
Cox 2020	7	480	6	442	1.5%	1.07 [0.36, 3.17]
Guo 2020	0	312	1	300	0.2%	0.32 [0.01, 7.84]
Subtotal (95% CI)		792		742	1.7%	0.95 [0.34, 2.65]
Total events	7		7			
Heterogeneity: $Tau^2 = 0.00$; $Chi^2 = 0.50$, df = 1 (P = 0.48); $I^2 = 0\%$						
Test for overall effect: Z = 0.10 (P = 0.92)						
**Total (95% CI)**		9768		9575	100.0%	0.94 [0.82, 1.07]
Total events	348		370			
Heterogeneity: $Tau^2 = 0.00$; $Chi^2 = 3.77$, df = 10 (P = 0.96); $I^2 = 0\%$						
Test for overall effect: Z = 0.92 (P = 0.36)						
Test for subgroup differences: $Chi^2 = 0.00$, df = 1 (P = 0.98), $I^2 = 0\%$						

Risk Ratio
M-H, Random, 95% CI

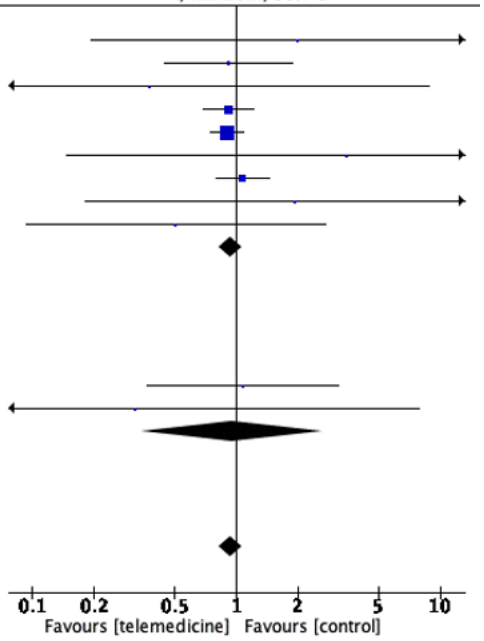

C. All-cause death

Study or Subgroup	Telemedicine Events	Telemedicine Total	Control Events	Control Total	Weight	Risk Ratio M-H, Random, 95% CI
**1.11.1 Randomized trials**						
Ayutthaya 2018	0	25	1	25	0.3%	0.33 [0.01, 7.81]
Fitzmaurice 1996	2	40	2	9	0.8%	0.23 [0.04, 1.39]
Fitzmaurice 2002	0	23	1	26	0.3%	0.38 [0.02, 8.78]
Matchar 2010	152	1465	157	1457	60.9%	0.96 [0.78, 1.19]
Poller 1993	1	122	0	64	0.3%	1.59 [0.07, 38.37]
Poller 2008	70	6605	62	6447	23.4%	1.10 [0.78, 1.55]
Sidhu 2001	0	41	4	48	0.3%	0.13 [0.01, 2.34]
Staresinic 2006	13	98	9	94	4.2%	1.39 [0.62, 3.09]
Zhu 2021	0	360	2	361	0.3%	0.20 [0.01, 4.16]
Subtotal (95% CI)		8779		8531	90.8%	0.98 [0.83, 1.17]
Total events	238		238			
Heterogeneity: $Tau^2 = 0.00$; $Chi^2 = 7.53$, df = 8 (P = 0.48); $I^2 = 0\%$						
Test for overall effect: Z = 0.18 (P = 0.86)						
**1.11.3 Cluster trials**						
Cox 2020	23	480	17	442	7.2%	1.25 [0.67, 2.30]
Fitzmaurice 2000	1	49	2	99	0.5%	1.01 [0.09, 10.87]
Guo 2020	3	312	8	300	1.6%	0.36 [0.10, 1.35]
Subtotal (95% CI)		841		841	9.2%	0.87 [0.39, 1.95]
Total events	27		27			
Heterogeneity: $Tau^2 = 0.17$; $Chi^2 = 2.81$, df = 2 (P = 0.25); $I^2 = 29\%$						
Test for overall effect: Z = 0.33 (P = 0.74)						
Total (95% CI)		9620		9372	100.0%	0.99 [0.84, 1.16]
Total events	265		265			
Heterogeneity: $Tau^2 = 0.00$; $Chi^2 = 10.34$, df = 11 (P = 0.50); $I^2 = 0\%$						
Test for overall effect: Z = 0.17 (P = 0.86)						
Test for subgroup differences: $Chi^2 = 0.08$, df = 1 (P = 0.77), $I^2 = 0\%$						

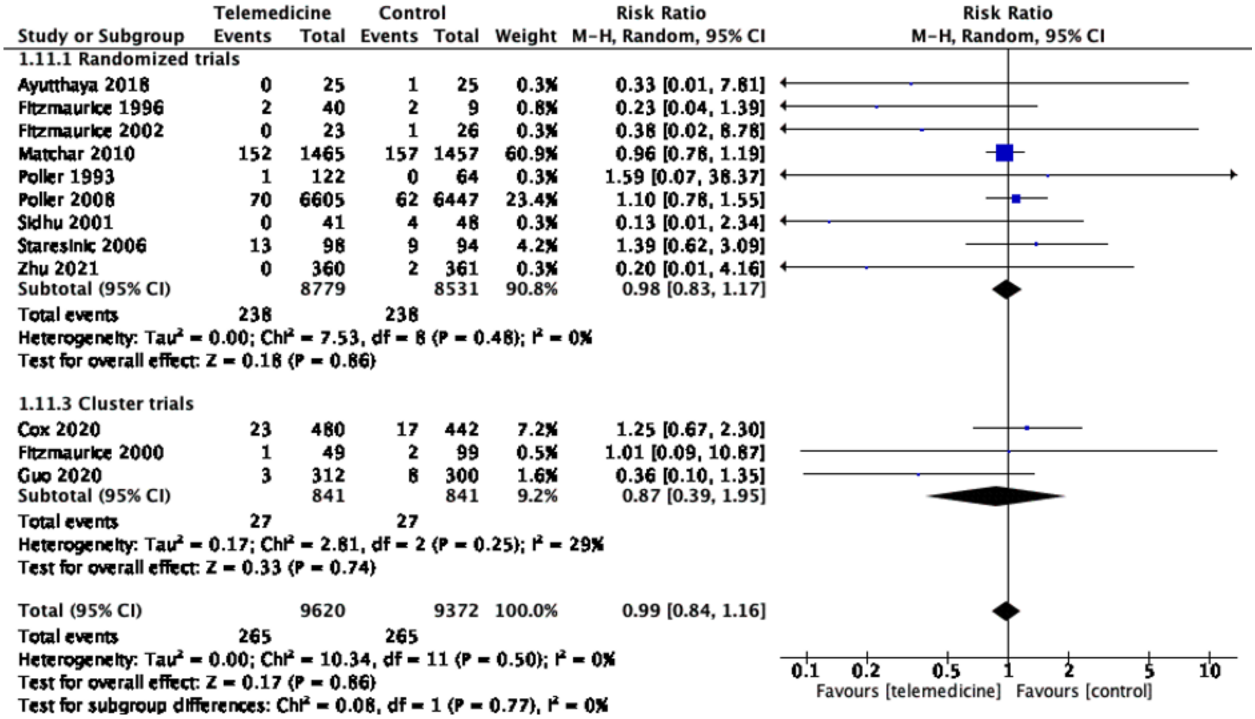

D. Time in therapeutic range

Study or Subgroup	Telemedicine Mean	Telemedicine SD	Telemedicine Total	Control Mean	Control SD	Control Total	Weight	Mean Difference IV, Random, 95% CI
**1.14.1 Randomized trials**								
Ayutthaya 2018	49.8	34.3	25	28	27.5	25	1.5%	21.80 [4.57, 39.03]
Borgman 2012	77.7	11.3	13	70.3	17.9	13	2.9%	7.40 [−4.11, 18.91]
Christensen 2011	79.9	2.22	83	72.7	2.35	40	11.4%	7.20 [6.33, 8.07]
Fitzmaurice 2002	74	16.1875	23	77	24.7581	26	2.9%	−3.00 [−14.59, 8.59]
Gadisseur 2003	67.7	15.9	99	64.7	22.1	221	8.2%	3.00 [−1.28, 7.28]
Khan 2004	71.1	14.5	39	70.4	24.5	40	4.2%	0.70 [−8.15, 9.55]
Matchar 2010	66.2	14.2	1465	62.4	17.1	1457	11.3%	3.80 [2.66, 4.94]
Nieuwlaat 2012	71	23.2	650	71.9	22.9	648	10.2%	−0.90 [−3.41, 1.61]
Poller 1998	63.3	28	137	53.2	27.7	148	6.0%	10.10 [3.63, 16.57]
Poller 2008	65.9	16.5	6605	64.7	17	6447	11.5%	1.20 [0.63, 1.77]
Rasmussen 2012	52.1	18.1	37	55	11.1	17	4.8%	−2.90 [−10.76, 4.96]
Staresinic 2006	57.8	39.1	98	55.1	39.1	94	3.1%	2.70 [−8.36, 13.76]
Vadher 1997a	60.7	25.6186	37	51.6	25.6186	44	3.0%	9.10 [−2.10, 20.30]
Vadher 1997b	67.6	25.7228	50	70.1	25.7228	46	3.4%	−2.50 [−12.80, 7.80]
Vogeler 2020	58	28	15	78	14	15	1.7%	−20.00 [−35.84, −4.16]
Zhu 2021	53	24	360	46	21	361	9.3%	7.00 [3.71, 10.29]
Subtotal (95% CI)			9736			9642	95.5%	3.22 [0.89, 5.54]
Heterogeneity: $Tau^2 = 11.55$; $Chi^2 = 167.25$, df = 15 (P < 0.00001); $I^2 = 91\%$								
Test for overall effect: Z = 2.71 (P = 0.007)								
**1.14.2 Cluster trials**								
Fitzmaurice 2000	69	10.4445	49	62	40.111	99	4.5%	7.00 [−1.43, 15.43]
Subtotal (95% CI)			49			99	4.5%	7.00 [−1.43, 15.43]
Heterogeneity: Not applicable								
Test for overall effect: Z = 1.63 (P = 0.10)								
Total (95% CI)			9785			9741	100.0%	3.38 [1.12, 5.65]
Heterogeneity: $Tau^2 = 11.47$; $Chi^2 = 168.07$, df = 16 (P < 0.00001); $I^2 = 90\%$								
Test for overall effect: Z = 2.93 (P = 0.003)								
Test for subgroup differences: $Chi^2 = 0.72$, df = 1 (P = 0.40), $I^2 = 0\%$								

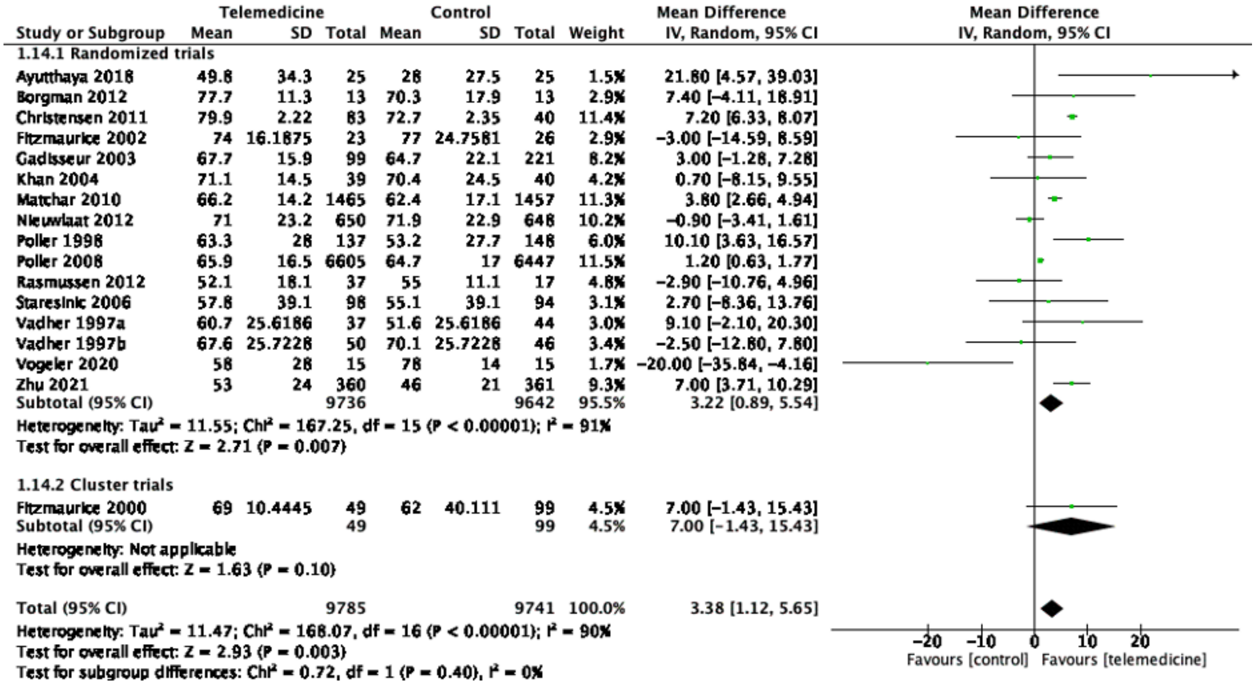

Figure S2: Forest plots of the comparison: Telemedicine interventions versus usual care. Sensitivity analysis with cluster studies data adjusted using an intracluster correlation coefficient of 0.05. Outcomes: A. Total thromboembolic events B. Major bleeding C. Death D. Time in therapeutic range.
